# Author Correction: Turn-key mapping of cell receptor force orientation and magnitude using a commercial structured illumination microscope

**DOI:** 10.1038/s41467-021-25577-5

**Published:** 2021-09-16

**Authors:** Aaron Blanchard, J. Dale Combs, Joshua M. Brockman, Anna V. Kellner, Roxanne Glazier, Hanquan Su, Rachel L. Bender, Alisina S. Bazrafshan, Wenchun Chen, M. Edward Quach, Renhao Li, Alexa L. Mattheyses, Khalid Salaita

**Affiliations:** 1 Wallace H. Coulter Department of Biomedical Engineering, Georgia Institute of Technology and Emory University, Atlanta, GA USA; 2 Department of Chemistry, Emory University, Atlanta, GA USA; 3 Aflac Cancer and Blood Disorders Center, Children’s Healthcare of Atlanta, Atlanta, GA USA; 4 Department of Pediatrics, Emory University School of Medicine, Atlanta, GA USA; 5 Department of Cell, Developmental, and Integrative Biology, University of Alabama at Birmingham, Birmingham, AL USA

**Keywords:** Polarization microscopy, Super-resolution microscopy, Nanoscale biophysics, Biosensors, Integrins

Correction to: *Nature Communications* 10.1038/s41467-021-24602-x, published online 3 August 2021.

The original version of this Article omitted the following from the Acknowledgements:

J.M.B., A.T.B., and R.G. acknowledge NSF GRFP grant no. 1444932. J.M.B. acknowledges NCI fellowship grant no. F99CA234959. A.T.B. acknowledges NCI fellowship grant no. F99CA245789. A.V.K. acknowledges NIH grant no. F31 F31CA243502. M.E.Q. acknowledges NIH grant no. F31 F31HL134241. R.L. acknowledges NIH grant no. HL082808. A.L.M. and K.S. acknowledge NIH grant no. R01GM131099. A.L.M. acknowledges NSF CAREER1832100. K.S. acknowledges NIH grant no. R01GM124472.

We thank Laura Fox-Goharioon, Neil Anthony, and William Giang of the Integrated Cellular Imaging Core Facility at Emory University for maintaining and troubleshooting the SIM microscope used in this work. We also thank Eric Rentchler of the Biomedical Microscopy Core at the University of Michigan for coordinating access to software for SIM super-resolution reconstructions. We thank Rong Ma of Emory University for providing graphical illustrations. Finally, we thank Tejeshwar Rao, Reena Beggs, Tomasz Nawara, and Will Dean of the Mattheyses lab at the University of Alabama, Birmingham for helpful conversations.

This has now been corrected in both the PDF and HTML versions of the Article.

