# Supplementary material for: Turn-key mapping of cell receptor force orientation and magnitude using a commercial structured illumination microscope
Source: Nat Commun. 2021 Aug 3;12:4693. doi: 10.1038/s41467-021-24602-x (PMC8333341; doi:10.1038/s41467-021-24602-x)
Supplement: Supplementary file 1 — Supplementary Information [file 41467_2021_24602_MOESM1_ESM.pdf]

Supplementary Information for:

Turn-key mapping of cell receptor force orientation and magnitude using a commercial structured illumination microscope

Aaron Blanchard[1], J. Dale Combs[2], Joshua M. Brockman[1], Anna V. Kellner[1], Roxanne Glazier[1], Hanquan Su[2], Rachel L. Bender[2], Alisina Bazrafshan[2], Wenchun Chen[3,4], M. Edward Quach[3,4], Renhao Li[3,4], Alexa L. Mattheyses[5], & Khalid Salaita[1,2]

[1]Wallace H. Coulter Department of Biomedical Engineering, Georgia Institute of Technology and Emory University, Atlanta, Georgia 30322, USA
[2]Department of Chemistry, Emory University, 1515 Dickey Drive, Atlanta, Georgia 30322, USA
[3]Aflac Cancer and Blood Disorders Center, Children's Healthcare of Atlanta,
[4]Department of Pediatrics, Emory University School of Medicine
[5]Department of Cell, Developmental, and Integrative Biology, University of Alabama at Birmingham, Birmingham, Alabama, 35294, USA
[*]Address correspondence to: k.salaita@emory.edu

Contents

I. Supplementary Figures 3

Supplementary Figure 1: Verification of linear polarization of excitation laser..... 3

Supplementary Figure 2: Analysis of DiI-doped SLB-coated microspheres imaged using SIM microscope. 4

Supplementary Figure 3: Azimuthal angle maps of 15 representative platelets. 6

Supplementary Figure 4: Platelets exhibit two distinct zones of force orientation. 7

Supplementary Figure 5: Comparison of Background Illumination Profile Correction Methods 8

Supplementary Figure 6: SIM vs. widefield (WF) resolution comparison 9

Supplementary Figure 7: Image resolution estimation using parameter-free decorrelation analysis 11

Supplementary Figure 8: Widefield (WF) and super-resolution images and linescans of 3T3 fibroblasts 12

Supplementary Figure 9: Validation of SIM acquisitions with SIMcheck 14

Supplementary Figure 10: Results of Monte Carlo simulations of force orientation measurement. 16

Supplementary Figure 11: Extended analysis of Monte Carlo simulation results. 17

Supplementary Figure 12: Analysis of photon counts 18

Supplementary Figure 13: Dependence of measured $\langle\theta\rangle$ on measured ϕ values. 19

Supplementary Figure 14: Quantification of tension area 20

Supplementary Figure 15: Quantification of the alignment parameter and primary axis of contraction 21

Supplementary Figure 16: Representative platelet timelapse data 22

Supplementary Figure 17: Simulated photobleaching suggests that increasing alignment is likely not a photobleaching-induced artifact. 23

Supplementary Figure 18: Measurement of experimental photobleaching and expected effect on results 24

Supplementary Figure 19: Comparison of SIM-MFM signal produced by T-cell receptor and platelet integrin forces 26

II. Supplementary Table 1 – Oligonucleotide sequences 28

III. Supplementary Table 2 – Comparison of high resolution TFM and MTFM techniques 29

IV. Supplementary Note 1 – Derivation of equations for analytical orientation measurement 30

V. Supplementary Note 2 – Examination of the effect of photobleaching on dynamic measurements of alignment 32

VI. Supplementary Note 3 – Description of analysis of T-cell receptor SIM-MFM data 33

VII. REFERENCES 34

I. Supplementary Figures

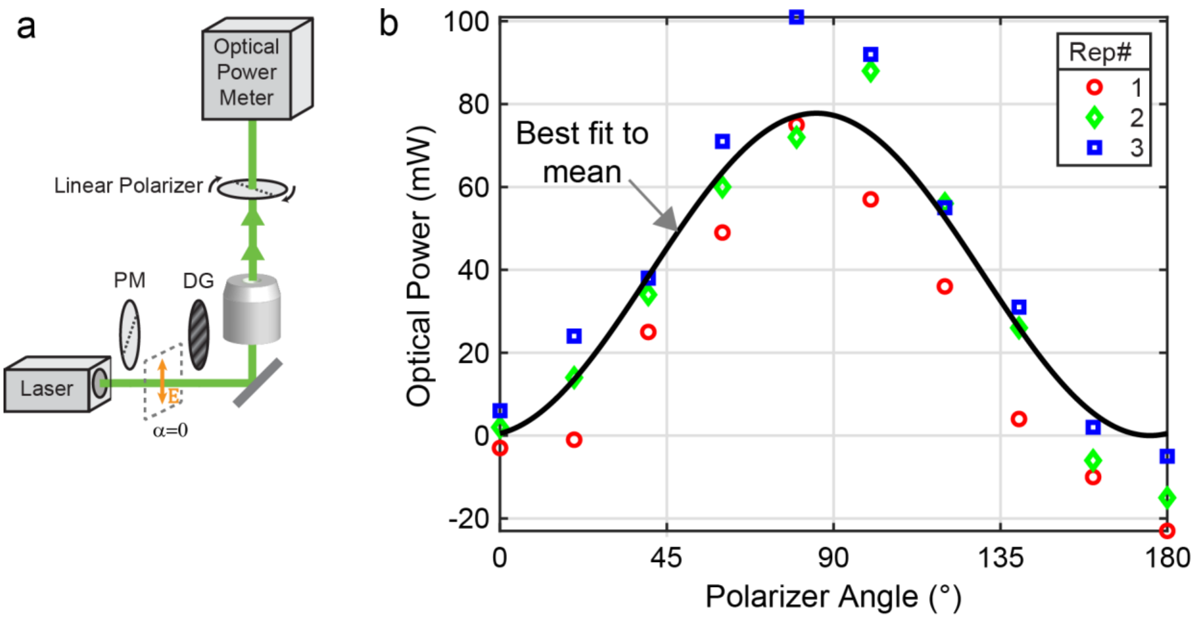

Supplementary Figure 1: Verification of linear polarization of excitation laser

Polarization verification was performed as described in the section paragraph of the methods subsection "**Structured illumination microscopy (SIM)**". **a)** Microscope excitation path diagram adapted from **Fig. 1a** depicting arrangement of the optical power meter and rotatable linear polarizer. The polarization modulator (PM) and diffraction grating (DG) used to create the SIM striped interference pattern were removed from the light path for this measurement. **b)** Plots of three experimental replicate measurements at various linear polarizer angles. Black curve shows best-fit $\sin^2$ curve fit to the triplicate averages. Fitting was performed using MATLAB's built-in fmincon function, and was constrained to have non-negative sinusoid minimum. Accordingly, the best-fit sinusoid had a minimum of zero, corresponding to linearly-polarized light. Elliptically polarized light would have produced a positive sinusoid minimum.

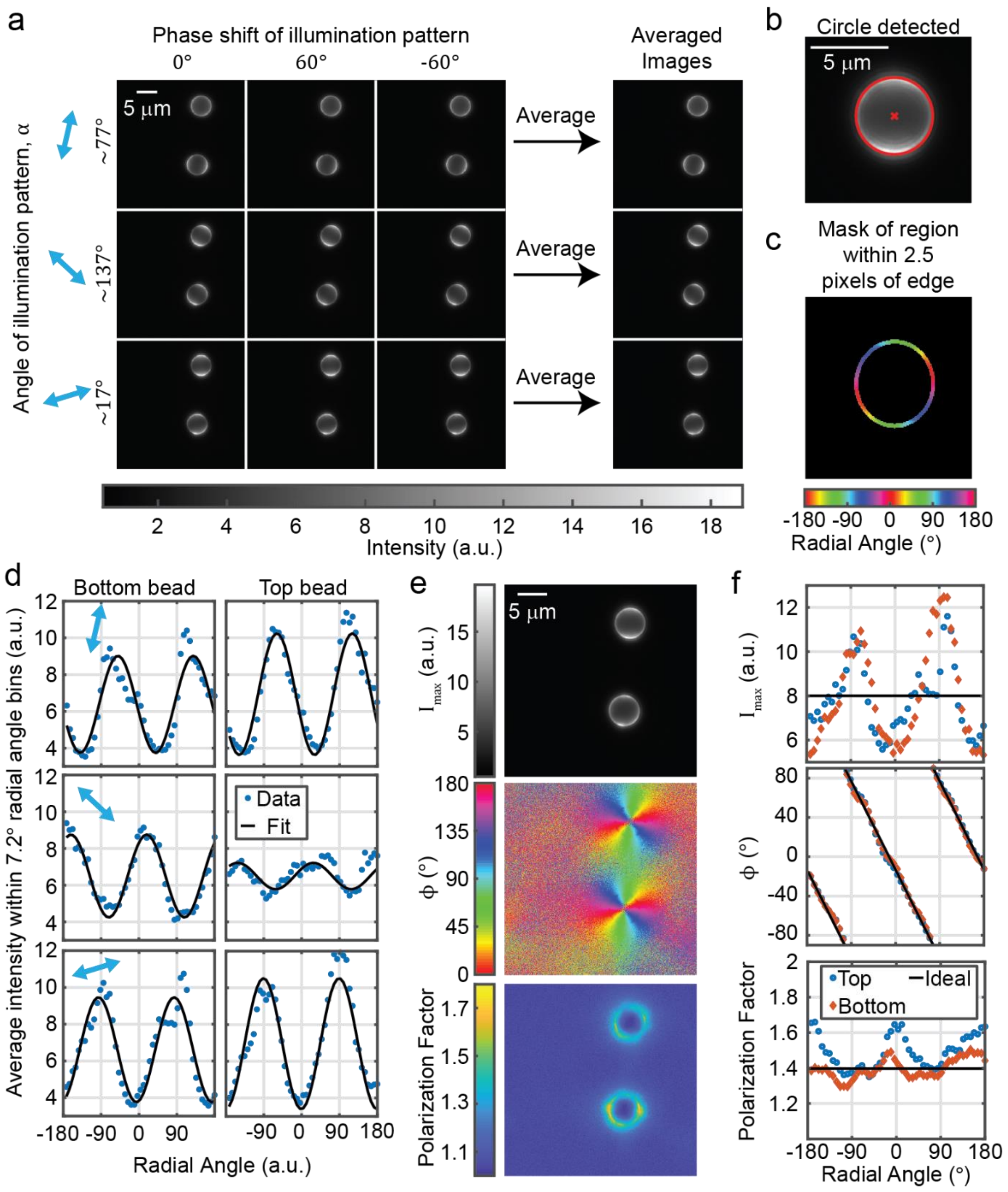

Supplementary Figure 2: Analysis of DiI-doped SLB-coated microspheres imaged using SIM microscope.

a) Raw 2D SIM images of DiI-doped supported lipid bilayer (SLB)-coated silica microspheres. **b)** Automatic detection of spheres using MATLAB's imfindcircles() command. **c)** Mask used for subsequent analysis. Mask is colored by the radial angle, which is used as a coordinate for subsequent analysis (in this

scenario, ϕ should be equal to the radial angle). **d)** fluorescence intensity as a function of radial angle for each of the two beads in the image. Each blue point represents the average value within one of 50 bins. A best-fit sinusoid is shown in each plot (black). **e)** I_{max}, ϕ, and the polarization factor ($(A + c)/c$) calculated for every pixel in the image. **f)** Quantities in **e** calculated as a function of radial angle (as in **d**). I_{max} and the polarization factor should not exhibit variations with respect to radial angle, do the variations shown likely reflect artifacts due to the index of refraction mismatch between the bead and the surrounding solution. Independent experiments with SLB-coated beads were repeated three times with similar results.

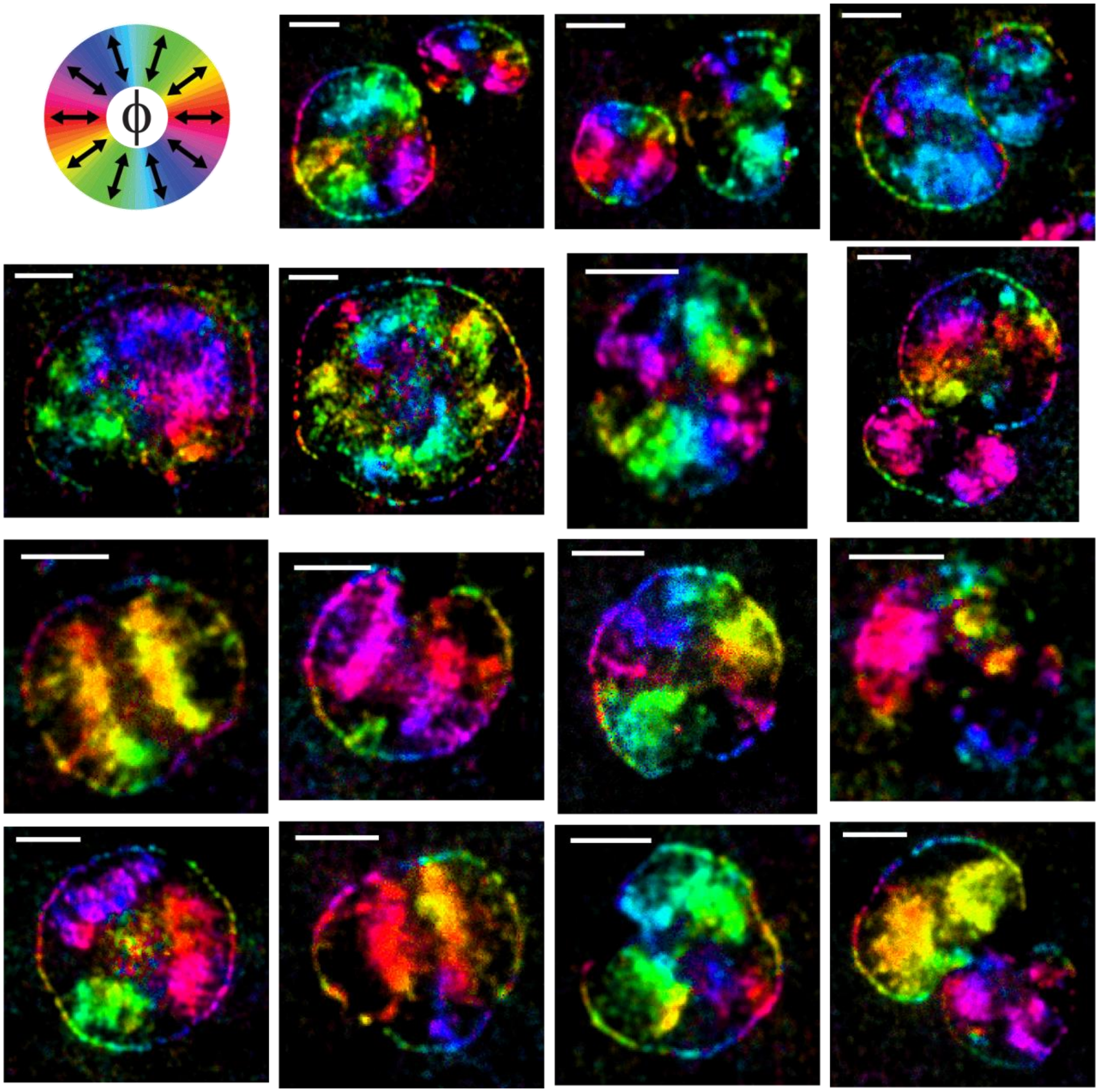

Supplementary Figure 3: Azimuthal angle maps of 15 representative platelets.

Human platelets were collected and imaged and the images were processed as described in the methods section. Each standard-resolution image shows a color-based ϕ map of one or two platelets. The scale bar for each image denotes 2 micrometers. Independent experiments with human platelets were repeated five times with similar results.

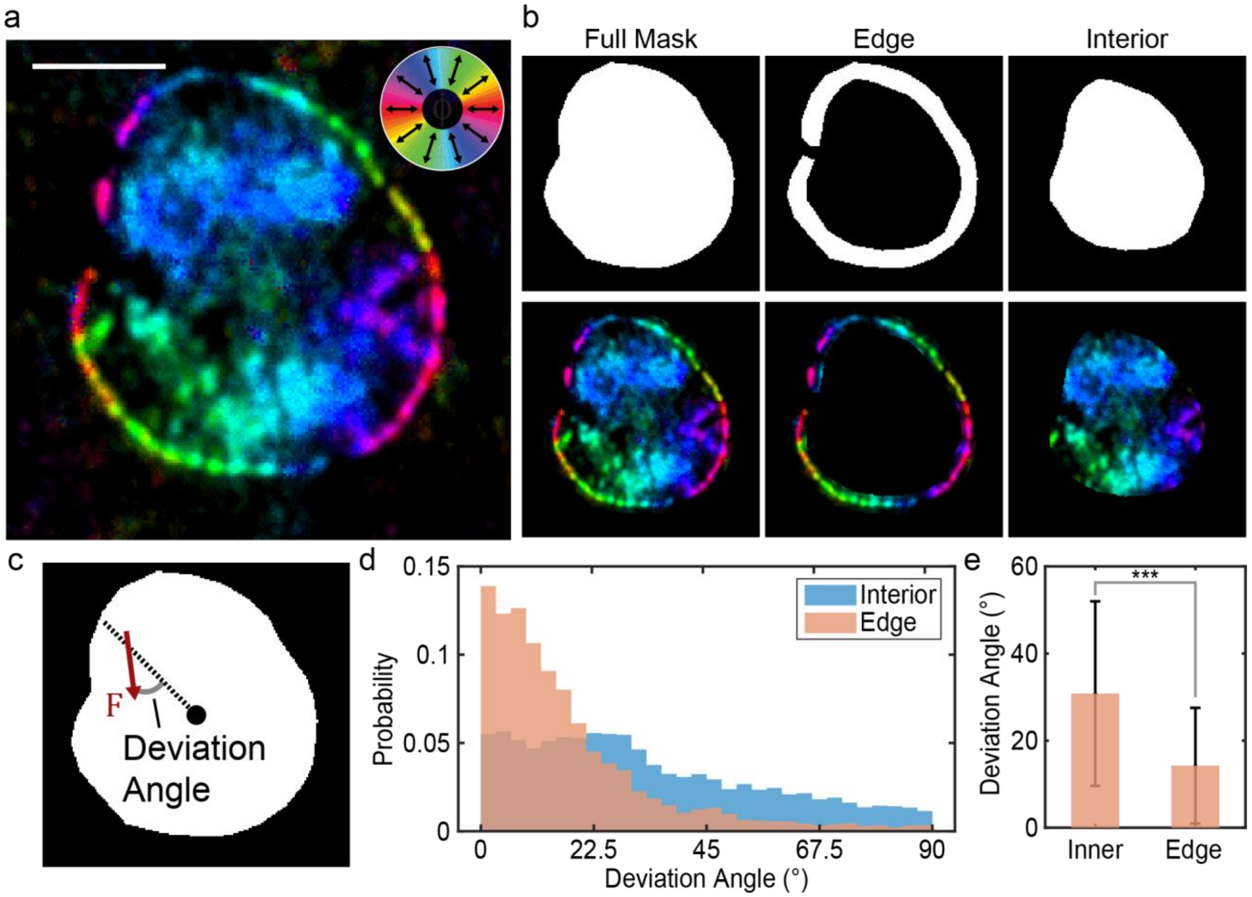

Supplementary Figure 4: Platelets exhibit two distinct zones of force orientation.

a) Colormap-based ϕ-map of a platelet. Scale bar denotes 2 micrometers. **b)** Hand drawn masks of the full cell, lamellipodial edge, and cell interior (top) with corresponding masked ϕ-maps (bottom). **c)** Depiction of "deviation angle" used to quantify the radiality of platelet integrin traction forces[1] (deviation angle for a given pixel is the angle between the force and the axis that links the mask's centroid to the pixel's centroid). **d)** Probability histogram of deviation angle in the interior and cell edge. **e)** Comparison of the average deviation angle of the two populations shown in **d** (errorbars show standard deviation). For this cell, there were n=5,757 interior pixels and n=11,572 edge pixels. *** denotes p<0.001, Wilcoxon two-sided rank-sum test. Independent experiments with human platelets were repeated five times with similar results.

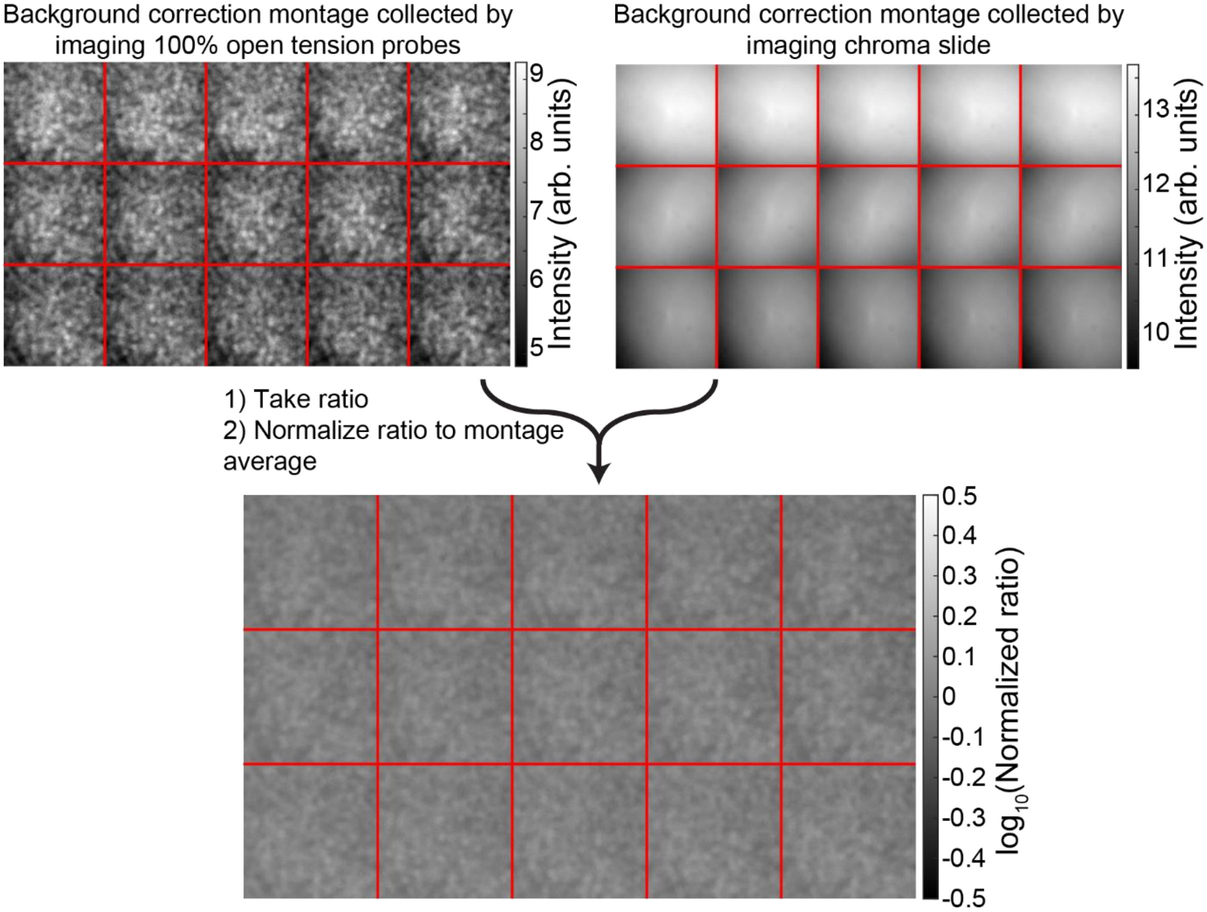

Supplementary Figure 5: Comparison of Background Illumination Profile Correction Methods

In this work, we used two different methods for correcting for variations in the illumination profile (see methods). To determine whether the two methods could be expected to produce similar results, we generated background illumination profiles using images collected on the same day for both methods (top). We then took the ratio of these two background illumination profiles. The resulting ratio (bottom) was essentially a flat field with no noticeable global variations. This analysis suggests that both methods are equally effective at accounting for long-range variations in intensity (i.e. the Gaussian illumination profile), as well as variations between individual images.

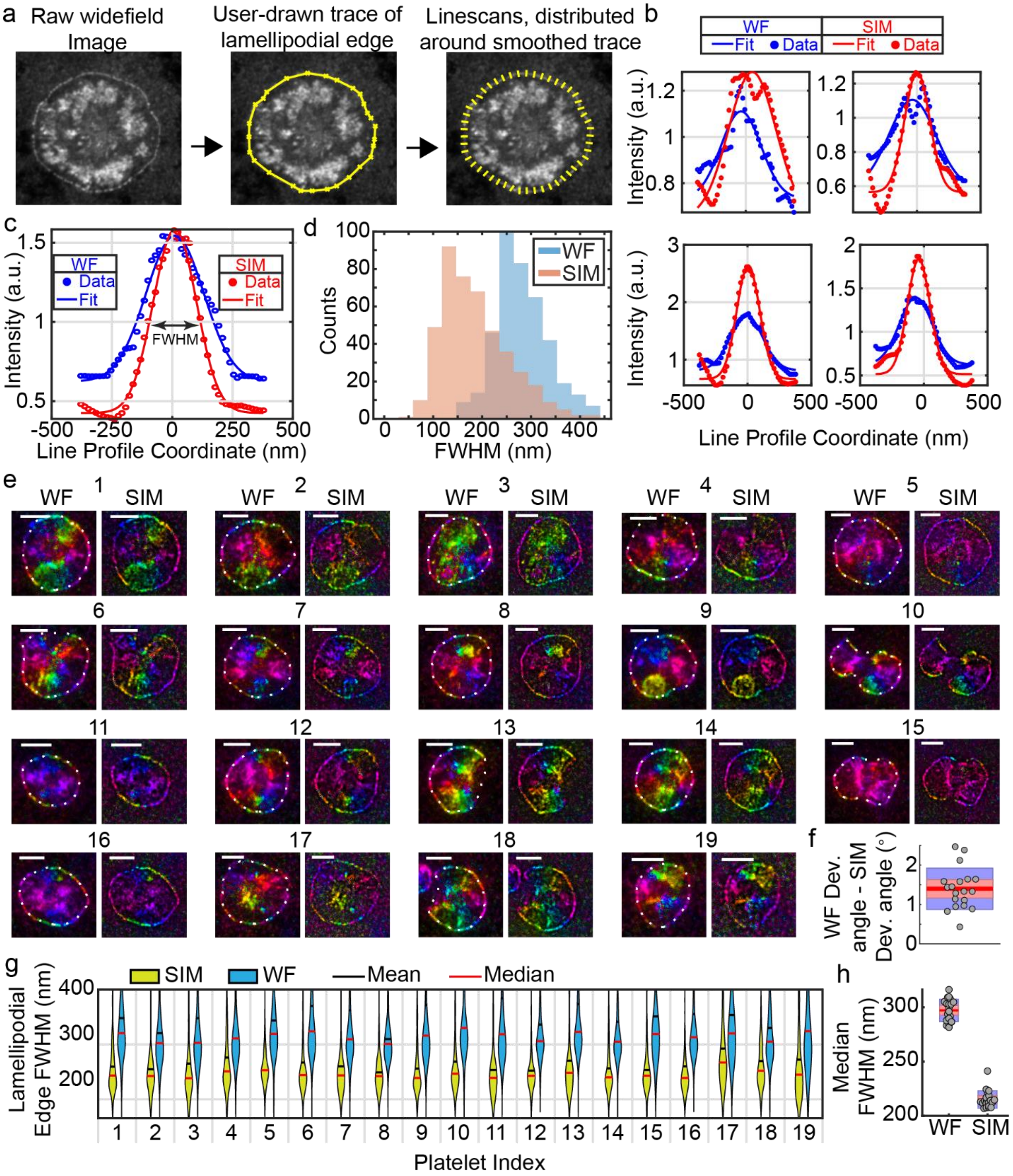

Supplementary Figure 6: SIM vs. widefield (WF) resolution comparison

a) Depiction of procedure used to obtain intensity linescans around the lamellipodial edge of the platelet. First, a polygon was traced around the platelet's perimeter using MATLAB's built-in getpts function. Then 500 points were interpolated around the perimeter of the closed polygon and the points were smoothed with a ten-point filter. At each of the 500 points, an intensity linescan perpendicular to the polygon's perimeter extending ~378 nm (6 WF pixels) in each direction was obtained using MATLAB's built-in improfile

function. **b)** A Gaussian function was then fit to each linescan using MATLAB's built-in "fit" function. Four representative linescans are shown, with both WF (blue) and SIM (red) shown. **c)** The full-width half maximum (FWHM) was then obtained from each Gaussian. **d)** Two histograms show the distribution of FWHM values recorded by repeating this process for each of the 500 linescans. Note that linescans were discarded if they did not meet all of the following conditions: either best-fit sinusoid's maximum is located at least 315 nm away from the linescan's midpoint; or either of the best-fit sinusoids has a maximum height of less than 0.2 (intensity units in these images are background-normalized). **e)** Nineteen pairs of SIM-MFM orientation colormaps are shown (for each pair, WF is on the left and SIM is on the right). Dotted white lines in each WF image show user-drawn and smoothed lamellipodial edge polygons used for generating linescans. White lines in top left corners of images denote scale bar of 2 µm. **f)** A boxplot showing the change in the average deviation angle (**Supplementary Fig 4**) of pixels within ~378 nm of the lamellipodial edge polygon when calculations are weighted using widefield MFM, rather than super-resolution reconstruction images. All platelets shown in **e** (n=19) are depicted in the boxplot. Each grey circle denotes the average deviation angle change from one of the platelets in the analysis. Red shading denotes standard error of the mean and purple denotes 95% confidence interval. We observe a small, but significant improvement of ~1.5 degrees when super-resolution reconstructions are used. **g)** Violin plots showing the distribution of FWHM values from linescans of SIM (yellow) and WF (blue) images. These plots show that the FWHM is consistently lower for SIM reconstructions compared to WF for all 19 platelets included in this analysis. For each violin plot, black and red horizontal lines denote population mean and median, respectively. **h)** Box plots showing the median FWHM values for all 19 platelets, showing an improvement of ~82 nm from ~295 nm to ~213 nm when super-resolution reconstructions are used. Red shading denotes standard error of the mean and purple denotes 95% confidence interval.

Under optimal conditions, the WF resolution of 561 nm light with NA=1.49 (as is the case with our microscope) is:

$$FWHM_{WF} = \frac{(0.51)(561\,nm)}{1.49} = 191\,nm$$

A two-fold improvement, as is generally expected for ideal implementations of SIM, would thus produce $FWHM_{SIM} = 96\,nm$, which is a resolution improvement of ~95 nm. As such, our implementation of SIM reconstruction with SIM-MFM, which yields an ~82 nm improvement in resolution, comes close to the ideal resolution enhancement. Independent experiments with human platelets were repeated five times with similar results.

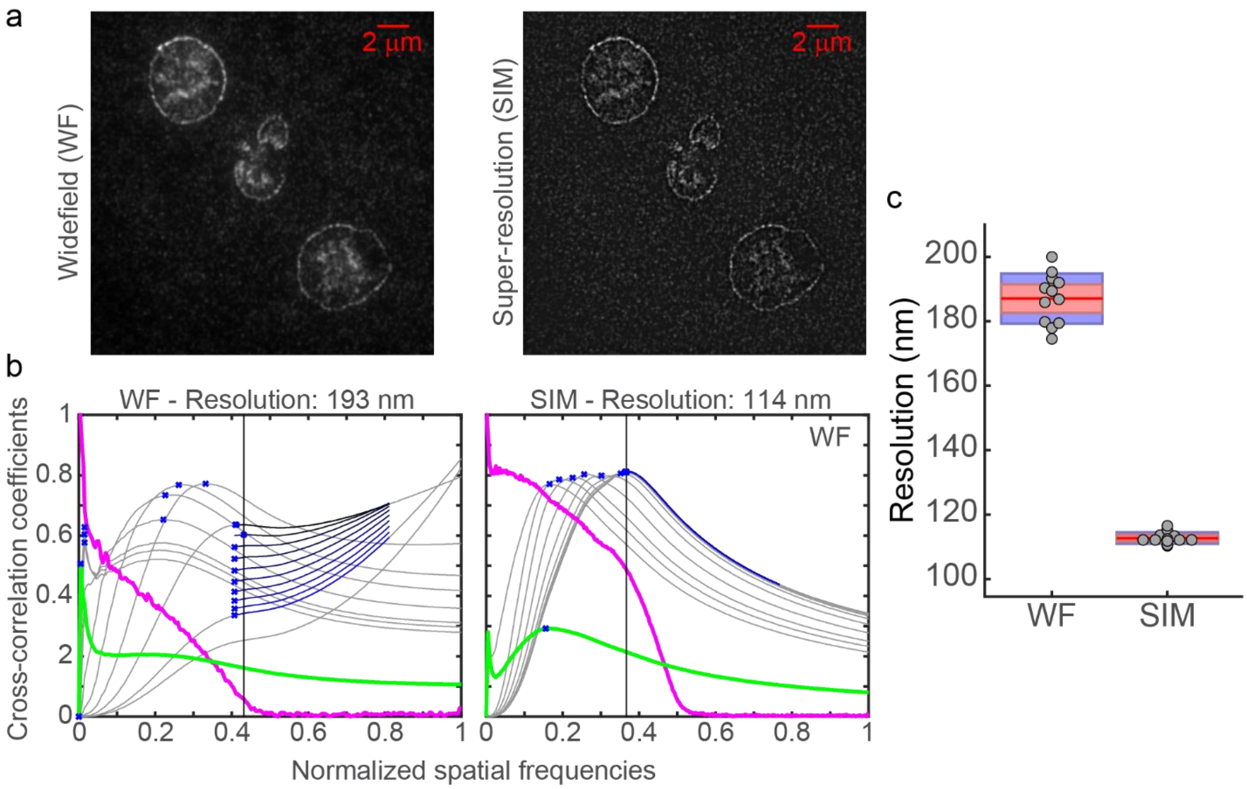

Supplementary Figure 7: Image resolution estimation using parameter-free decorrelation analysis

a) Representative SIM-MFM I_{max} images of human platelets, shown in widefield (left – a 512x512 image with 41.7 nm pixel size) and super-resolution (right – a 1024x1024 SIM reconstruction with 20.85 nm pixel size). **b)** Corresponding output plots of images shown in **a** processed using decorrelation analysis software[2]. According to ref. 4: green line denotes decorrelation functions before high-pass filtering; magenta line denotes the radial average of log of absolute value of Fourier transform of image; gray lines denote all high-pass filtered decorrelation functions; blue to black lines denote decorrelation functions with refined mask radius and high-pass filtering range; blue crosses denote all local maxima; vertical lines denote cut-off frequency. Resolution is 2 multiplied by the pixel size divided by the cutoff frequency. For more information, see ref. 4. **c)** Boxplots showing the estimated resolution of twelve pairs of images in widefield and super-resolution. Through this analysis, we observed a spatial resolution of $193 \pm 8\, nm$ (mean $\pm$ standard deviation) in widefield and $114 \pm 2\, nm$ in super-resolution. Independent experiments with human platelets were repeated five times with similar results.

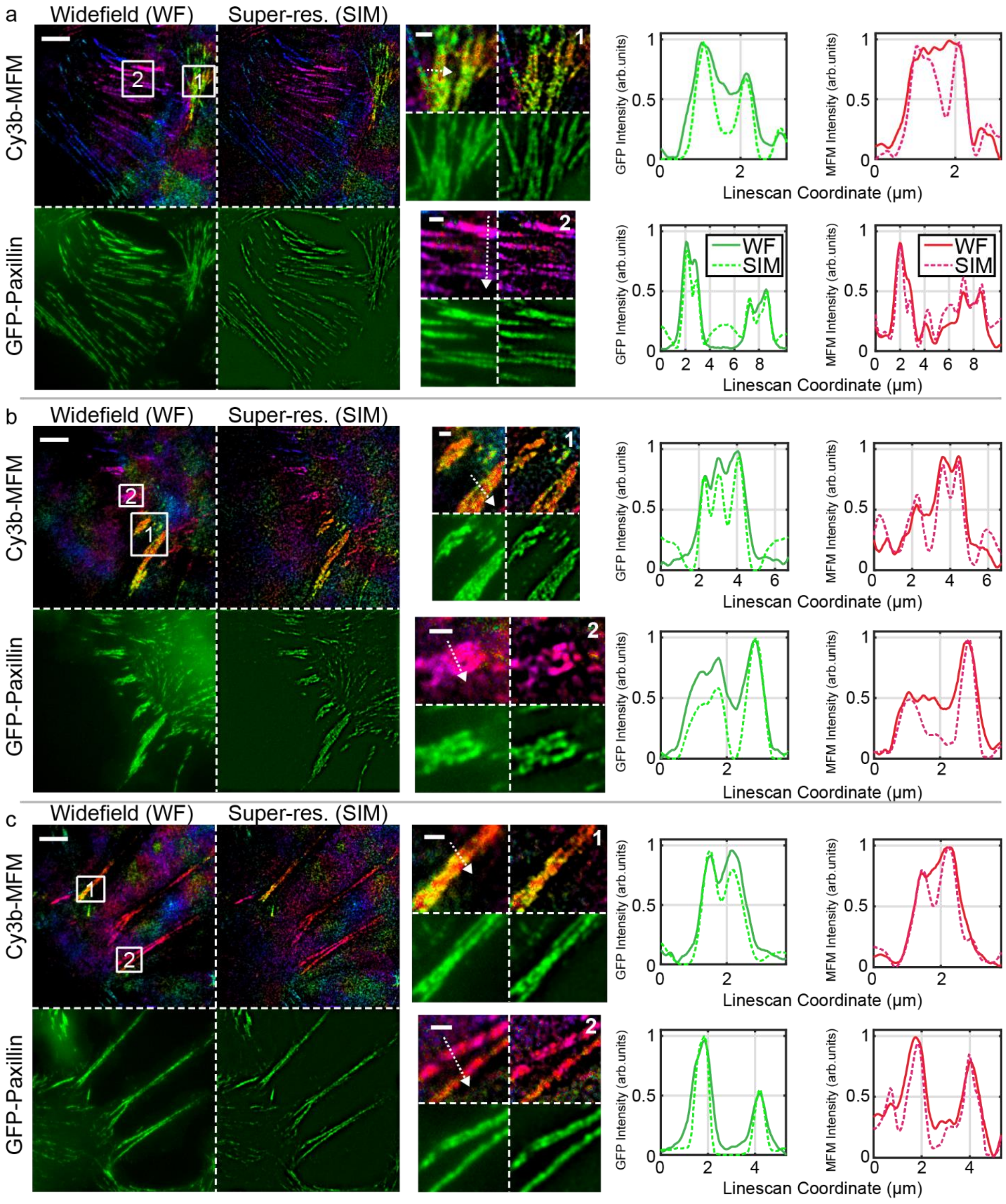

Supplementary Figure 8: Widefield (WF) and super-resolution images and linescans of 3T3 fibroblasts

a-c) Three subfigures each show representative data for one 3T3 fibroblast cell imaged using SIM-MFM. Four full images (left) show MFM ϕ orientation maps and GFP-paxillin images in widefield and super-resolution (scale bar: 5 μm). Two numbered, white rectangles in the WF MFM image are shown in zoom-

ins (middle) labeled with corresponding numbers in the top-right hand corner (scale bar: 1 µm). For each zoom-in, linescans (denoted by white dotted line in the WF MFM zoom-in) of all four images are shown in plots to the right. All linescans were obtained using MATLAB's built-in improfile function to sample user-drawn line segments at 100 evenly spaced points, minimum-subtracted, maximum-normalized, and smoothed via five-point averaging. Several of these linescans and zoom-ins show features that are only resolvable (or become sharpened) after applying SIM super-resolution reconstruction. Independent experiments with 3T3 fibroblasts were repeated three times with similar results.

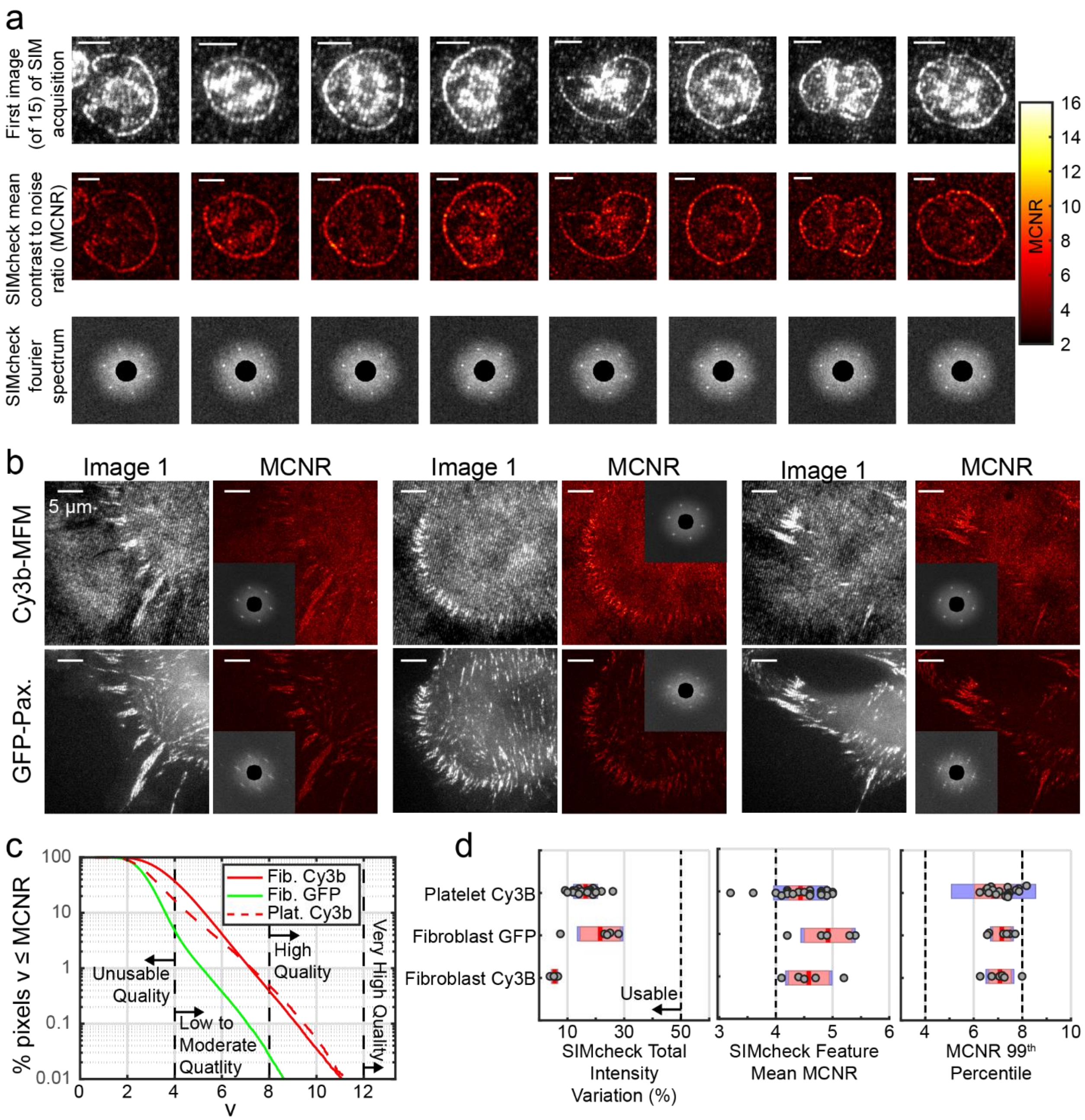

Supplementary Figure 9: Validation of SIM acquisitions with SIMcheck

To verify that SIM acquisitions were suitable for SIM reconstructions we analyzed images with the SIMcheck ImageJ plugin[3]. **A)** For each of nine representative platelets, the first Cy3 image of the fifteen image SIM-MFM acquisition (top – note the visible striping pattern), a heatmap of the modulation contrast to noise ratio (MCNR) output by the SIMcheck plugin (middle), and a Fourier spectrum output by the SIMcheck plugin (bottom) is shown. Scale bar: 2 µm. A colorbar on the right shows the range of expected MCNR values. MCNR is a metric of how strongly a pixel's intensity modulates due to phase shifting of the striping pattern, with higher MCNR indicating better reconstruction quality. According to guidelines laid out within SIMcheck's documentation, an MCNR>12 denotes excellent quality, MCNR between 8 and 12 denotes good quality, MCNR between 4 and 8 denotes low to moderate quality, and MCNR<4 is unusably

poor. The MCNR images show that bright signal in platelet SIM-MFM acquisitions generally falls in the 4-8 “low to moderate quality” range. Supporting this finding, Fourier spectra show six bright first-order spots around the central region (as is expected with SIM acquisitions such as these) but lack the farther-out second order spots that would generally be expected for high quality acquisitions. **b)** For each of three representative 3T3 fibroblasts, four images are shown: The first image of the 15-image SIM acquisition (left) and the MCNR heatmap (right – see the colorbar in **a**) are shown for the Cy3b MFM channel (top) and GFP-paxillin channel (bottom). Fourier spectra (generated by the SIMcheck plugin) are also shown as insets in each of the MCNR heatmaps. Note that very faint second order spots can be seen in these Fourier spectra. **c)** Plots showing the distribution of MCNR values on a logarithmic scale, in the format of one minus the cumulative density function. Cy3b-MFM acquisitions for both human platelets and 3T3 fibroblasts show >15% of pixels are within the usable quality range (which is reasonable considering the sparsity of tension signal) and the brightest ~0.3% of pixels have “good quality” MCNR values in the MCNR>8 range. GFP-Paxillin images show ~5% of usable pixels – the discrepancy between the two images likely arises due to the increased prevalence of bright, high MCNR pixels in the background of Cy3b-MFM acquisitions. **d)** Horizontal boxplots showing three different summary statistics for nineteen representative platelets and five representative 3T3 cells. The first two summary statistics – the total intensity variation (TIV – left) and the feature mean MCNR (middle) are produced by the SIMcheck plugin, while the third is created by taking the 99th quantile of MCNR heatmaps such as those shown in **a** and **b**. Solid red line indicates mean, dashed red line indicates median, red boxes show standard error of the mean and blue boxes shows 95% confidence interval of the mean. Grey circles indicate individual datapoints. Independent experiments with human platelets were repeated five times with similar results. Independent experiments with 3T3 fibroblasts were repeated three times with similar results.

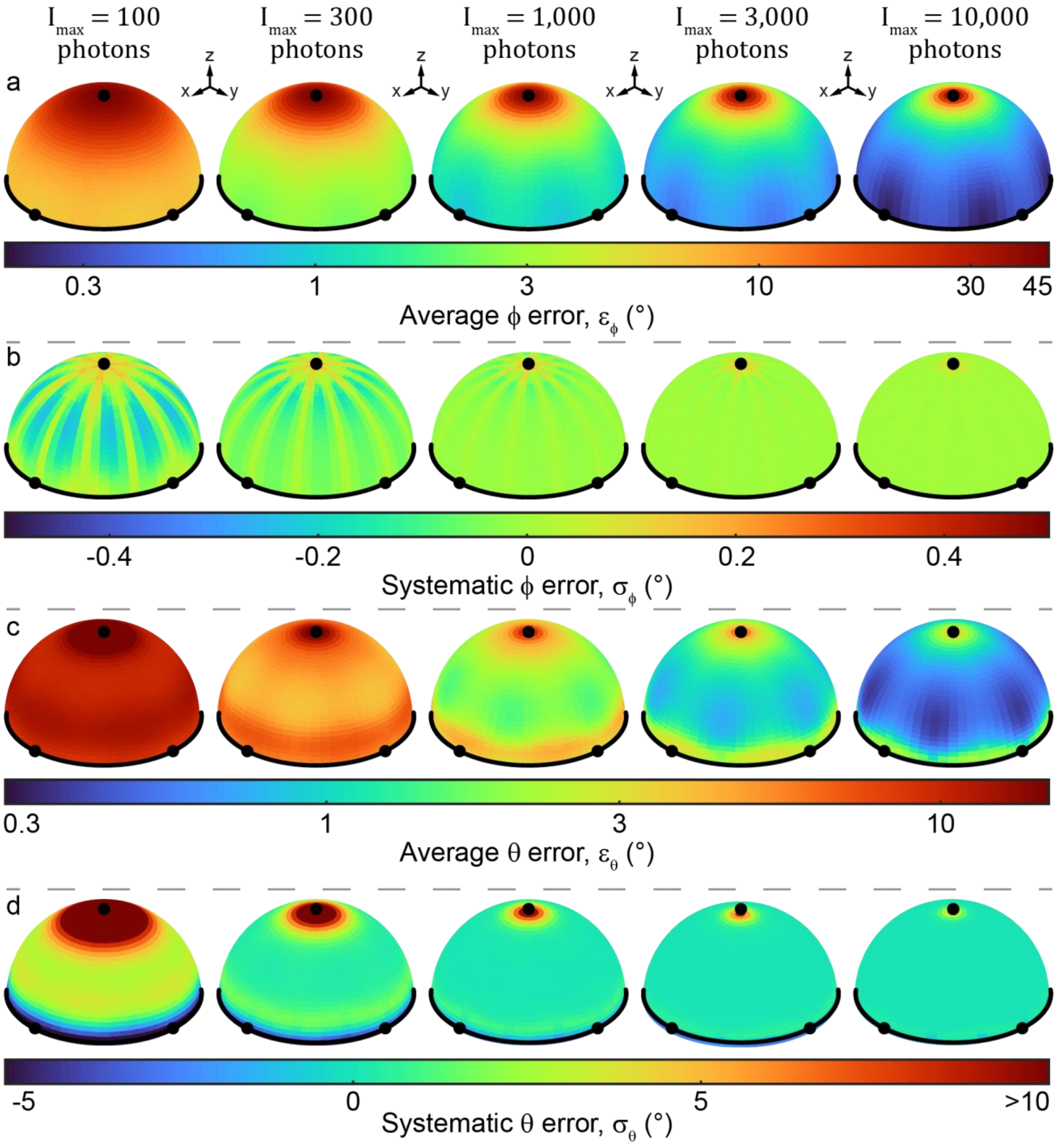

Supplementary Figure 10: Results of Monte Carlo simulations of force orientation measurement.

Several unit hemispheres are shown. Each hemisphere has three dots denoting the points where the x-, y-, and z-axes intersect with the hemispheres. A black curve also shows each hemisphere's intersection with the x-y plane. The color of each point on a hemisphere denotes the error of the orientation that passes through that point. The orientation- and photon count-dependence of four types of error are shown (see **Methods** for calculations): **a)** average ϕ error (ε_ϕ), **b)** systematic ϕ error (σ_ϕ), **c)** average θ error (ε_θ), and **d)** systematic θ error (σ_θ). Each is shown at five different signal-to-noise ratios.

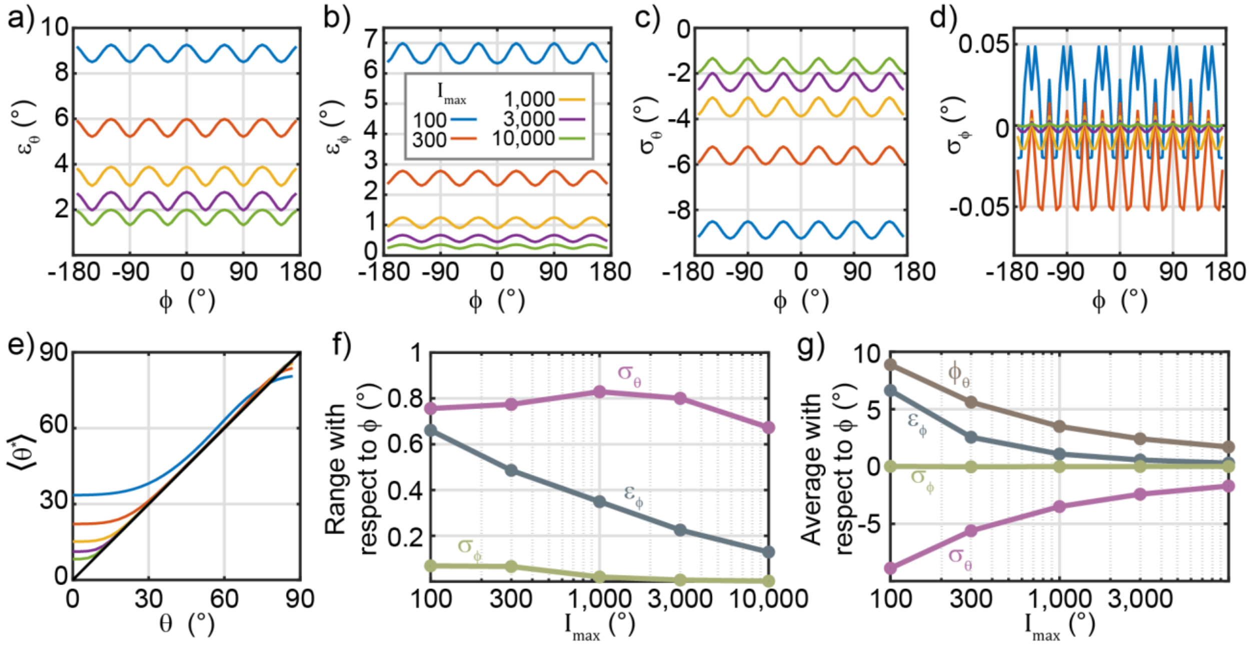

Supplementary Figure 11: Extended analysis of Monte Carlo simulation results.

a-d) ε_θ, ε_ϕ, σ_θ, and σ_ϕ as a function of ϕ with $\theta = 90°$ at five different signal-to-noise levels. These results show that all four metrics exhibit small periodic variations with respect to ϕ. **e)** The average measured θ value, $\langle\theta^*\rangle$, as a function of the true θ value. These results show a previously-observed[1] result: θ is biased systematically towards higher values when θ is very low because noise generally causes an increase in the best-fit sinusoid's amplitude. This effect decreases with increasing signal-to-noise. **f)** Range of curves shown in **a-d** showing the amplitude of the variation with respect to I_{max}. The curve for ε_θ is not shown because $\sigma_\theta = -\varepsilon_\theta$ when $\theta = 90°$, making the two range curves equivalent. **g)** Average of ε_θ, ε_ϕ, σ_θ, and σ_ϕ curves shown in **a-d** as a function of I_{max}, showing that all four types of error decrease with increasing signal-to-noise.

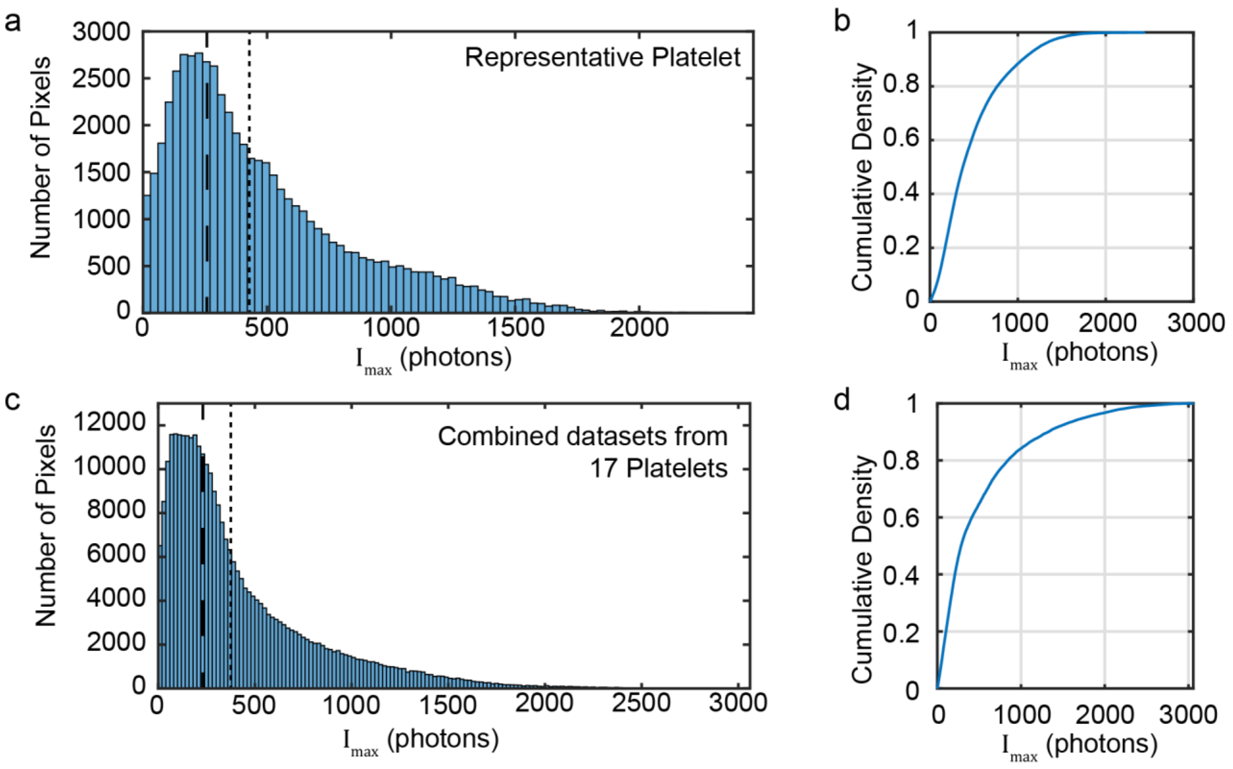

Supplementary Figure 12: Analysis of photon counts

Photon counts were estimated from arbitrary units (arb. Units) by 1) subtracting the 200 arb. Unit baseline from raw images, 2) multiplying by the pre-amplification factor (4.9, also called “Conversion Gain #1” in the nd2 image metadata), and 3) dividing by the conversion gain (100). We then used a masking procedure described in **Supplementary Fig 14** to select pixels that were included in SIM-MFM analyses. **A)** A histogram of photon counts following this full process is shown for an individual platelet, along with **b)** a cumulative density function of counts. **C,d)** Same as **a** and **b**, but for an aggregated dataset consisting of 17 platelets. The data shows that most accepted signal lies in the range of 300<I_{max} <1,000 photons, while the brightest 10% of signal lies in the range of 1,000<I_{max}<3,000 photons. Therefore, the MFM force orientation measurements are reliable based on the photon intensities collected.

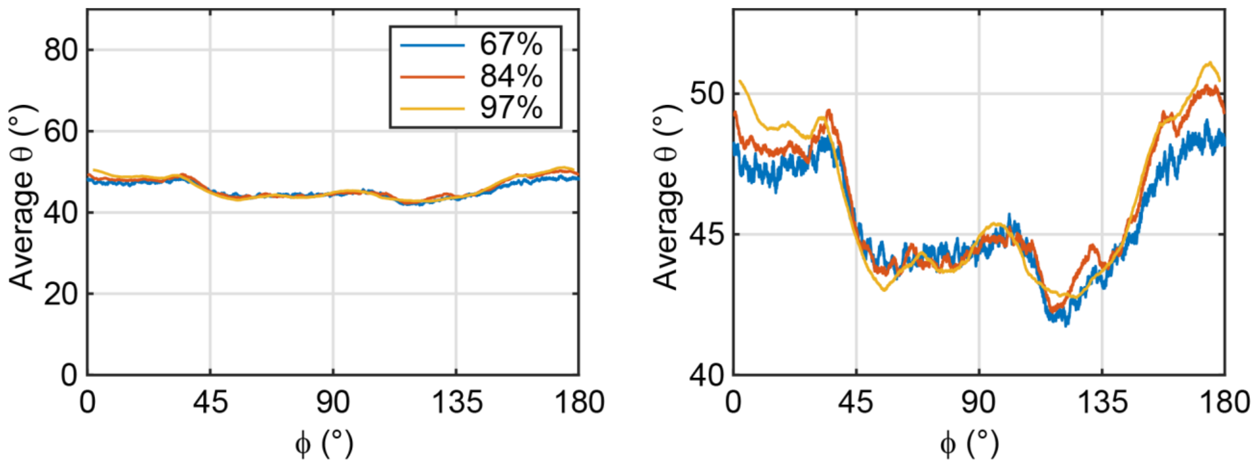

Supplementary Figure 13: Dependence of measured $\langle\theta\rangle$ on measured ϕ values.

A 500-point rolling average of θ values accumulated from n=37 platelet images as a function of ϕ. Plot is shown with the y-axis spanning the full range of potential θ values (left) and a zoom in on the curves (right). Three curves are shown, with thresholds cutoffs set as the 67th, 84th, and 97th percentiles of the accumulated dataset's intensity values. The similarity between the curves shows that the slight variation with respect to ϕ is not an artifact of the selected signal threshold cutoff.

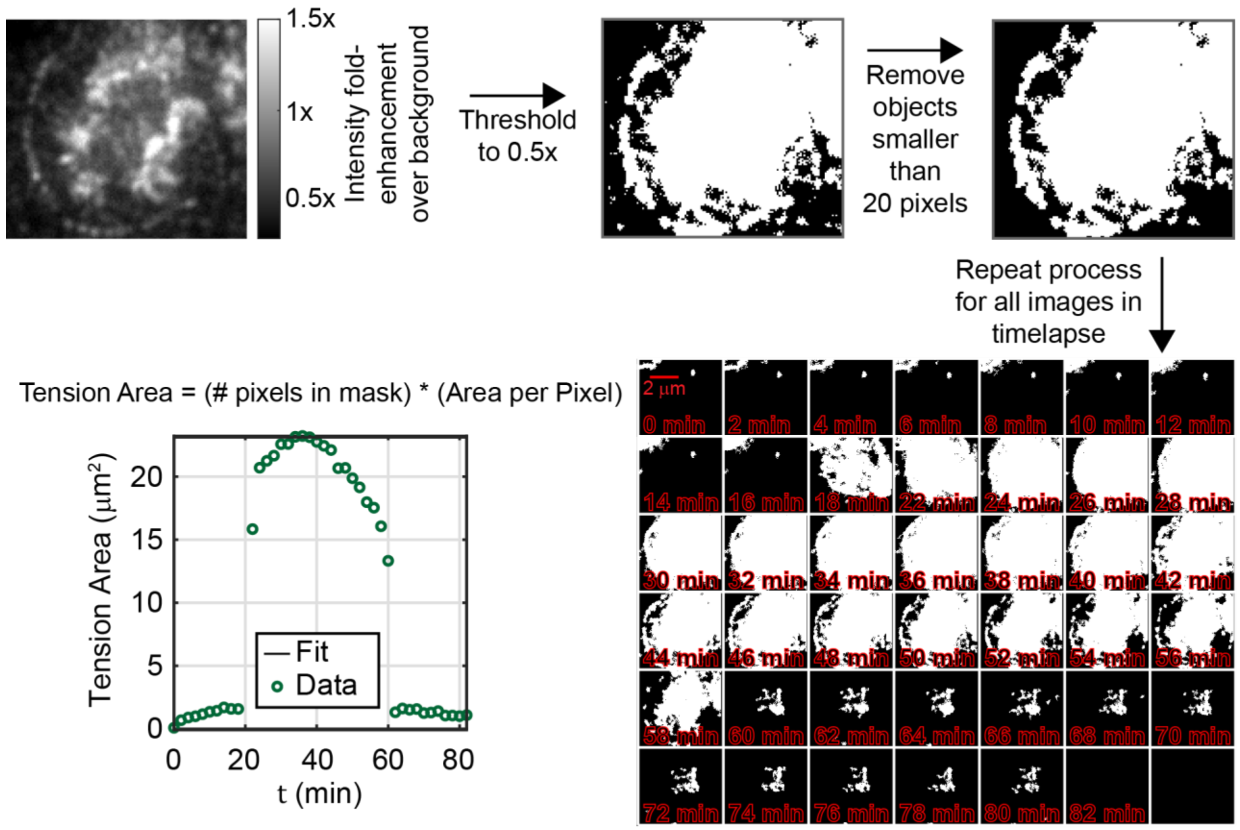

Supplementary Figure 14: Quantification of tension area

Tension area (T) was quantified using a thresholding-based process shown here. An I_{avg} image was first thresholded such that pixels with a >50% increase in intensity compared to background were kept. Then, Objects smaller than 20 pixels were removed. This simple two-step combination produces a mask that retains the vast majority of molecular tension signal, yet simultaneously discards cell-free background pixels with high fidelity. Tension area was then calculated by multiplying the number of pixels in the mask by the area per pixel (equal to $(41.7\ \text{nm})^2$). For timelapse quantification of tension area, this process was repeated on each image in the stack. Independent timelapse experiments with human platelets were repeated three times with similar results.

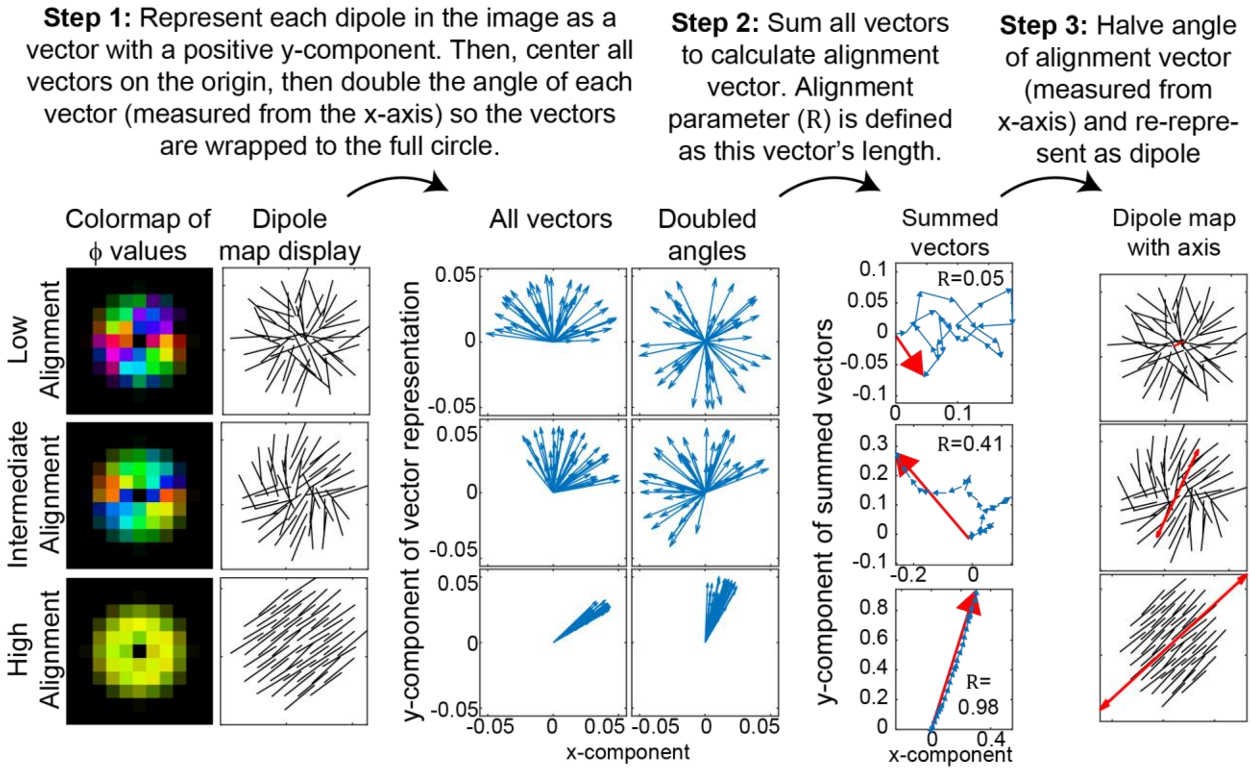

Supplementary Figure 15: Quantification of the alignment parameter and primary axis of contraction

The alignment parameter[4] (R) and the axis of contraction were determined via a process described in the figure.

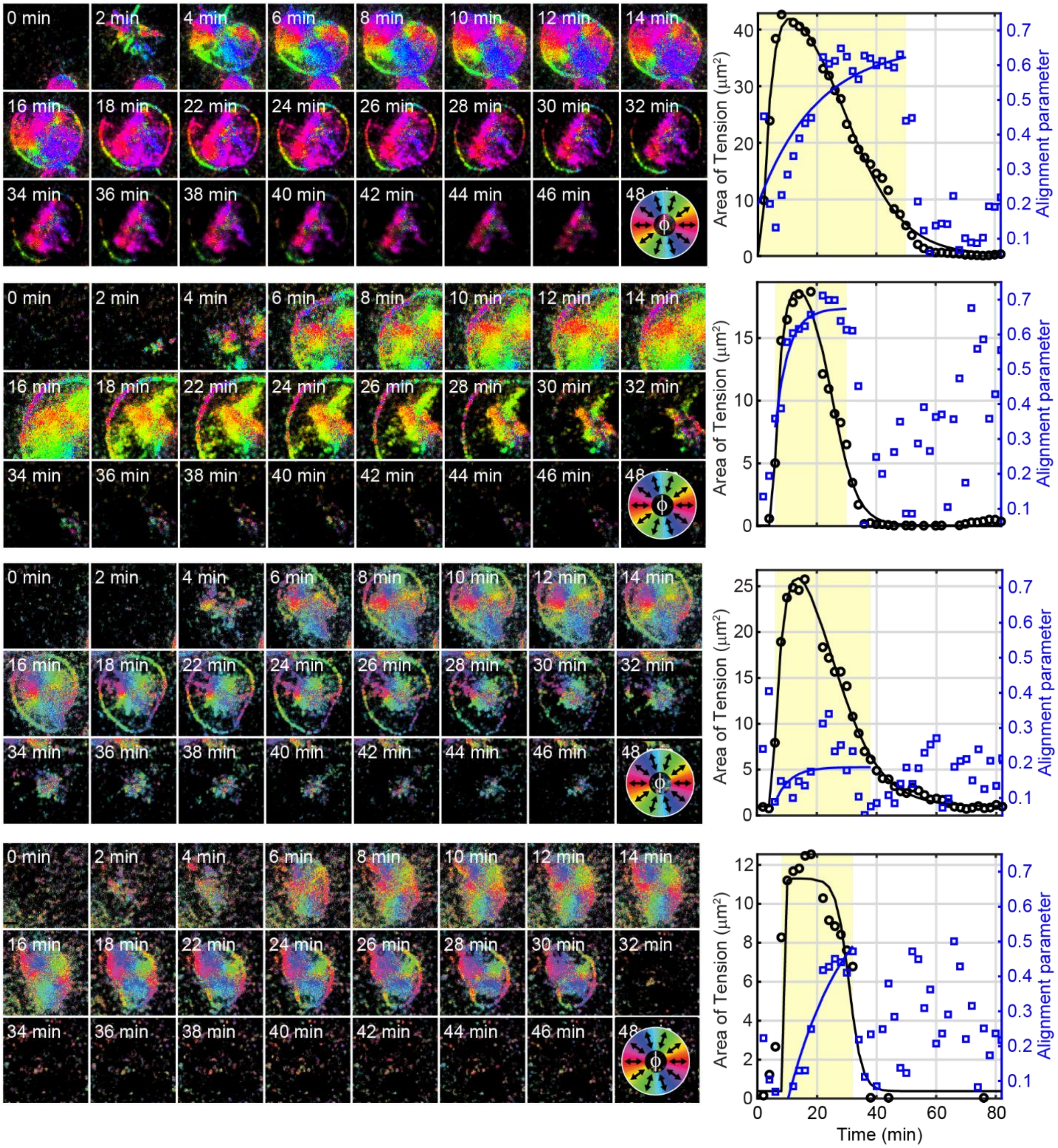

Supplementary Figure 16: Representative platelet timelapse data

Four example timelapses of platelets spreading on surfaces, exhibiting gradual increases in alignment, are shown in a similar format to **Fig. 5**. For each timelapse, colormap ϕ-maps are shown for 24 timepoints on the left and. On the right, tension area (black circles) and the calculated alignment parameter (blue circles) are plotted, each with a fit curve of corresponding color. The alignment parameter fit was only applied to the window shaded with yellow, which corresponds to the time of platelet adhesion. Independent timelapse experiments with human platelets were repeated three times with similar results.

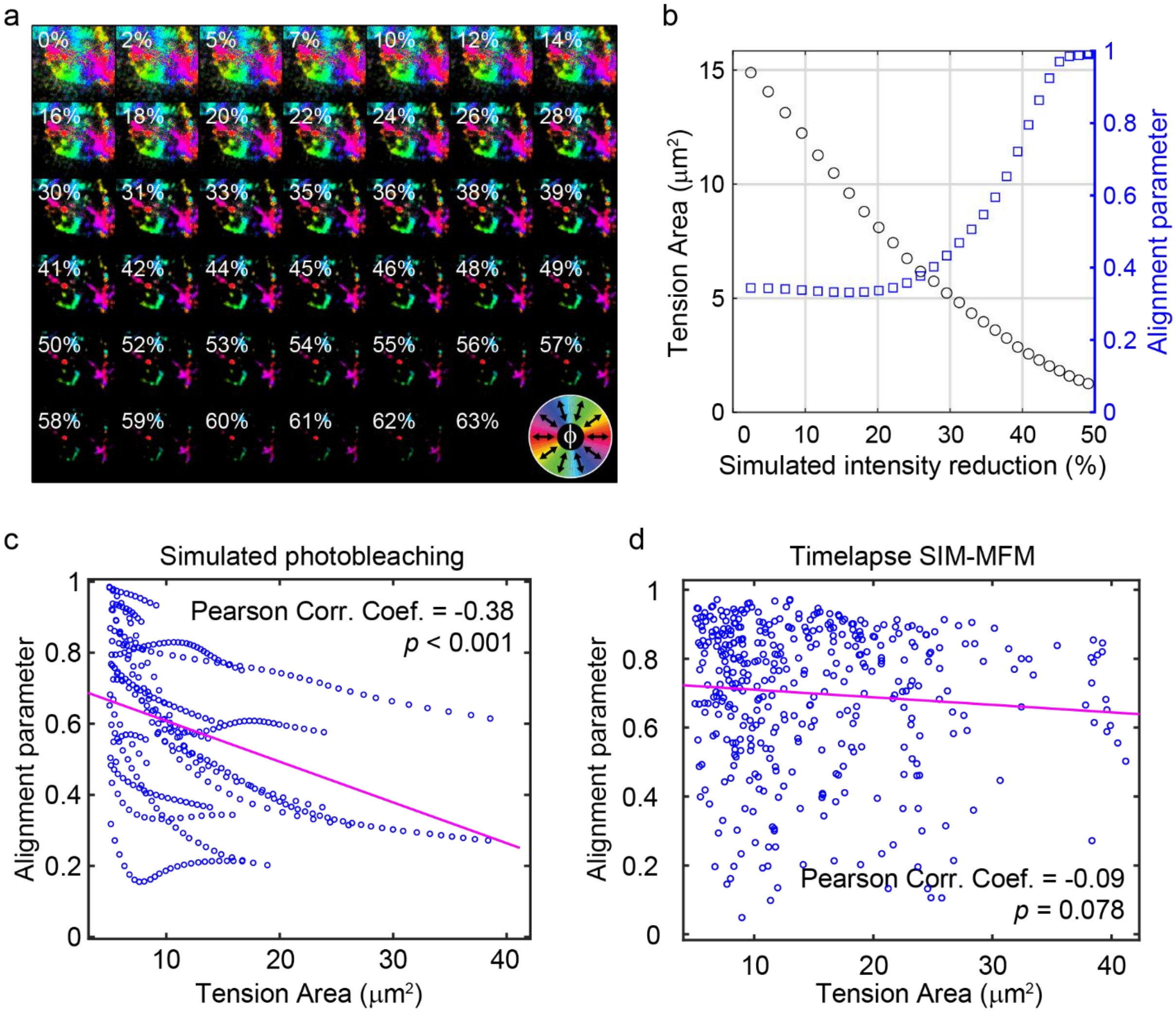

Supplementary Figure 17: Simulated photobleaching suggests that increasing alignment is likely not a photobleaching-induced artifact.

a) Simulated photobleaching as performed by gradually scaling the fluorescence intensity of an image. A montage of ϕ-colormap images is shown, each depicting the platelet shown in **Fig. 5a** at $t = 22\ min$ with its intensity reduced by an amount denoted in the image (e.g. 32% reduction). As the intensity is decreased, certain regions of the image fail to persist through the intensity cutoff and become excluded from the R and T calculations. **b)** Tension area (T) and the alignment parameter (R) measured as a function of the amount of simulated photobleaching for the representative platelet shown in **a**. While T decreases monotonically, R stays constant until about 25% intensity reduction, at which point it begins to increase to and eventually converge towards $R = 1$. **c)** A Pearson's correlation analysis of R and T, including data from simulated photobleaching of all 28 increasing-alignment platelets at their timepoint of maximum cumulative brightness, revealed a highly significant negative correlation between T and R. This analysis included only simulated photobleaching conditions where $T > 5\ \mu m^2$. The p-value (3.52×10^{-55}) is the output of the Pearson's Correlation Coefficient measurement performed with MATLAB's "corr" function. **d)** In contrast, a correlation analysis of all datapoints from the same 28 platelets at timepoints between t_{attach} and $t_{attach} + t_{detach}$ revealed no significant correlation between T and R. Therefore, it does not appear that photobleaching meaningfully results in artificially-increased R values.

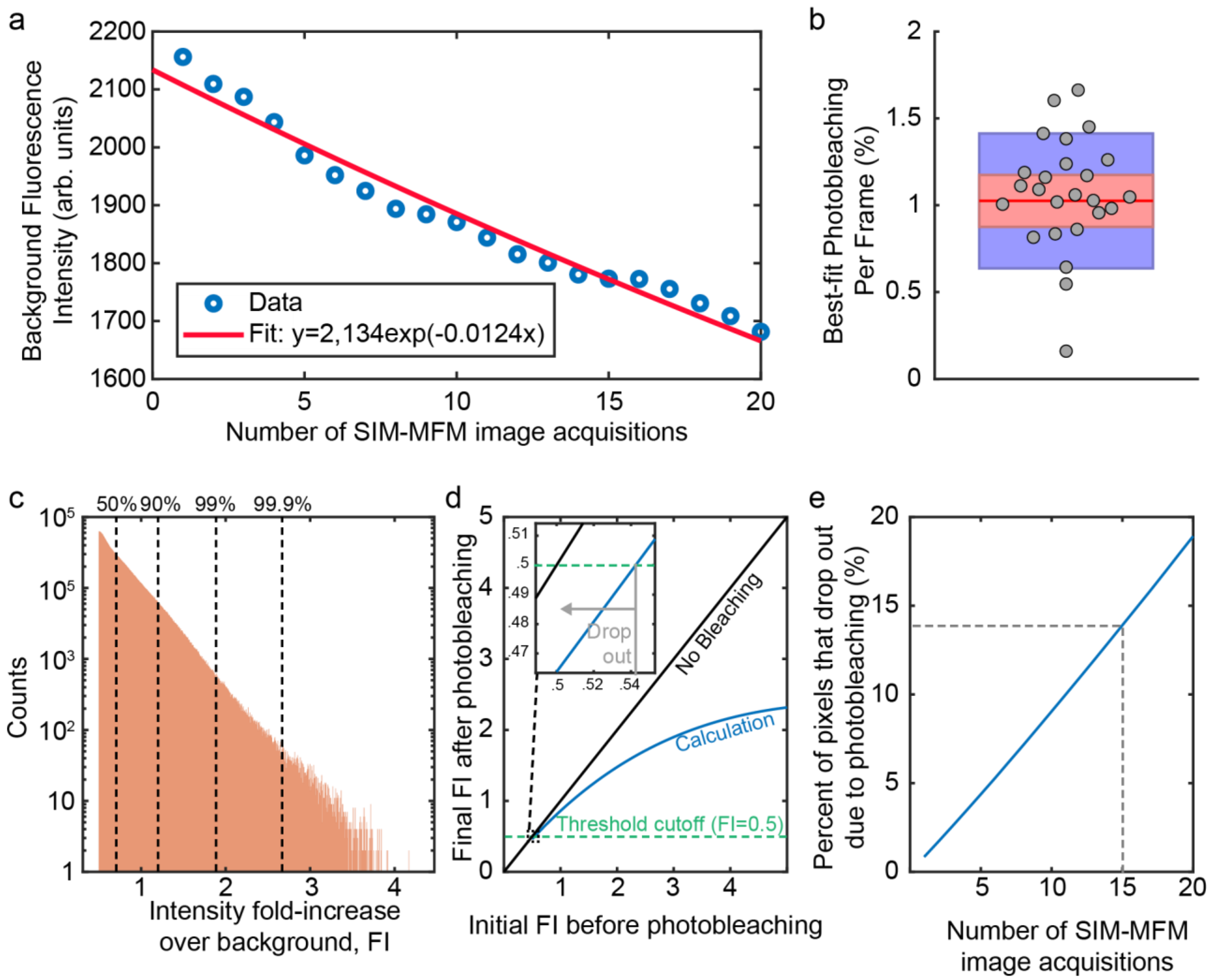

Supplementary Figure 18: Measurement of experimental photobleaching and expected effect on results

a) The fluorescence intensity of the quenched tension sensor background (with the 200 arb. unit camera baseline subtracted) was measured by averaging I_{avg} within a platelet-free region of a SIM-MFM timelapse (blue circles). Photobleaching was then quantified by fitting an exponential decay function to the average background intensity (red curve). The exponential decay rate constant of $k_{bleach,bkrd} = 0.0124$ corresponds to 1.24% photobleaching per SIM-MFM acquisition. **b)** Boxplot showing the best-fit percent photobleaching per SIM-MFM acquisition for 25 different timelapses (gray circles). An average (red line) of ~1% was measured. Red shading denotes standard error of the mean and purple denotes 95% confidence interval. **c)** Histogram (with logarithmic y-axis) of intensity fold-increase over background (FI) of all pixels included in the 81 masked platelet timelapses collected for this work. Vertical dotted lines denote the 50th, 90th, 99th, and 99.9th percentiles of FI. **d)** Plot of expected FI after photobleaching (FI_{after}) as a function of FI before photobleaching (FI_{before}) due to 15 full SIM-MFM acquisitions (blue curve). The threshold of $FI = 0.5$ used in the masking procedure is shown with a green dashed line, and a black line shows $FI_{after} = FI_{before}$. This calculation was performed using an estimate of 1% photobleaching of the quenched background per acquisition as shown in **b**. Opened tension probes are expected to bleach more rapidly than quenched probes, so the rate of photobleaching of opened tension probes can be calculated from the quenching efficiency (QE):

$$k_{bleach} = \frac{k_{bleach,bkrd}}{1 - QE}$$

If tension probes within a local region corresponding to a single pixel are considered from a population level and assumed to exchange between acquisitions (this assumption is reasonable because the integrin-ligand bond lifetime is generally known[5] to be on the order of 10 seconds and our images are taken at an interval of 2 minutes) can also be calculated from the quenching efficiency:

$$FI = \frac{I}{I_{bkrd}} - 1 = \frac{p_{open}}{1 - QE}$$

where p_{open} is the proportion of tension probes open within a given region and I_{bkrd} is the intensity of the fluorescent background. FI_{after} can be expressed as a function of p_{open}, k_{bleach}, and FI_{before}:

$$FI_{after} = FI_{before}\left(1 - \left(1 - p_{open}k_{bleach}\right)^{n}\right)$$

where n denotes the number of SIM-MFM acquisitions. This equation simplifies such that QE, which was previously measured as ~97% (ref. [6]), drops out of the equation:

$$FI_{after} = FI_{before}\left(1 - \left(1 - FI(1 - QE)\frac{k_{bleach,bkrd}}{1 - QE}\right)^{n}\right)$$
$$FI_{after} = FI_{before}\left(1 - \left(1 - k_{bleach,bkrd}FI\right)^{n}\right)$$

We used this equation to calculate FI_{after} as a function of FI_{before}. Our results show that pixels with higher FI_{before} exhibit larger extents of photobleaching, but only the dimmest pixels (those with FI_{before}<~0.54) transition below the $FI = 0.5$ cutoff due to photobleaching and drop out of the masking procedure. **e)** A plot of the percent of pixels that drop out (assuming a distribution of FI_{before} shown in **c**) of the masking procedure due to photobleaching as a function of n, showing a linear trend with a slope of 0.93% drop-out per image. Dotted line shows that at $n = 15$, ~14% of pixels drop out of the masking procedure due to photobleaching.

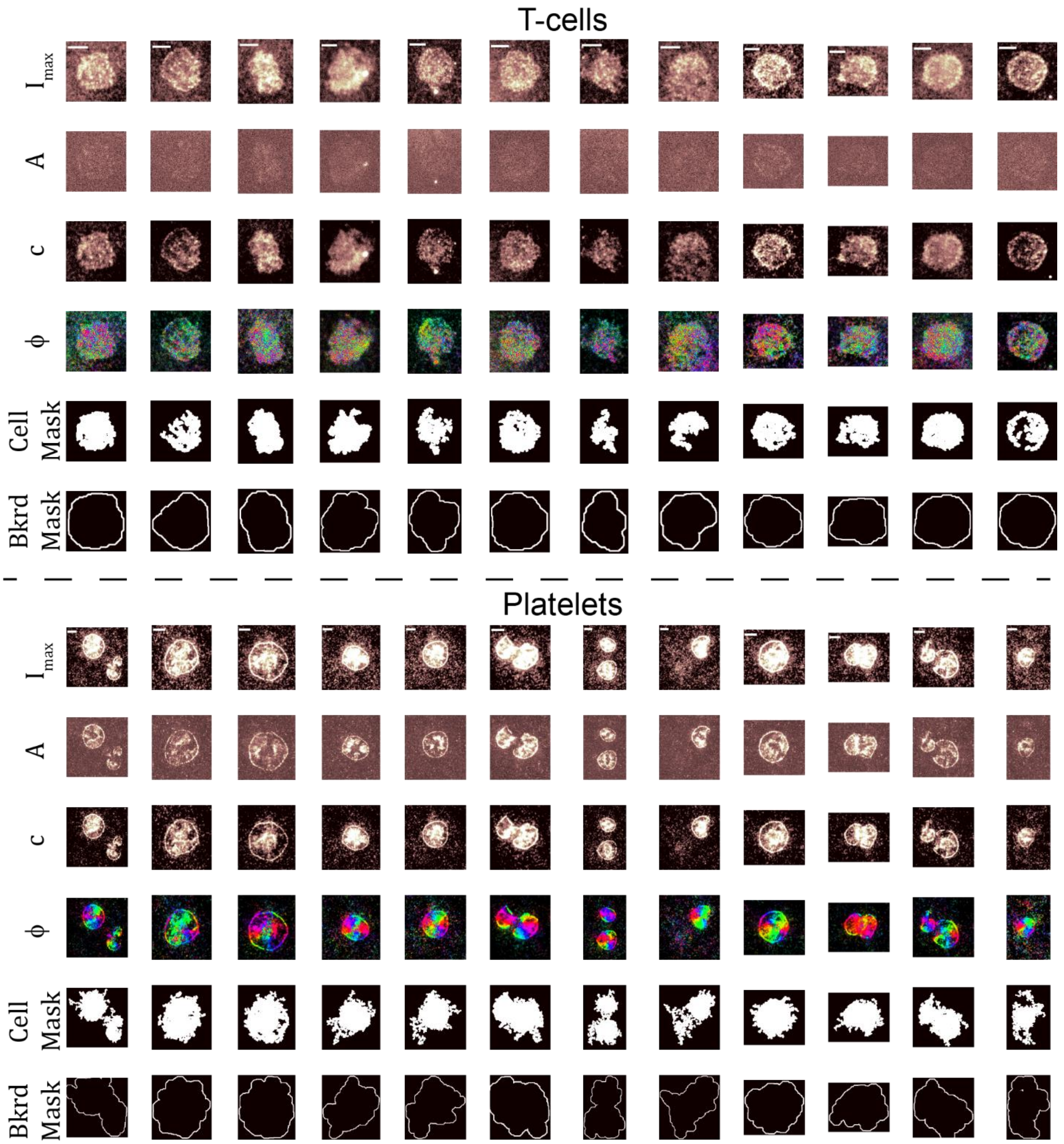

Supplementary Figure 19: Comparison of SIM-MFM signal produced by T-cell receptor and platelet integrin forces

This figure is divided in two sections that have the same structure. The top half of the figure shows representative T-cell data, while the bottom half shows representative data for platelets, which serve as a control. Each panel shows twelve representative cells (one in each platelet) and has 6 rows corresponding to I_{max}, A, c, ϕ, and the cell and background masks used to quantify $\langle A \rangle$ and $\langle c \rangle$ within the cells and the cell-free background. Notably, the T-cell A images show signal that is barely distinguishable from background, while platelet A images show substantially brighter signal than background. Additionally, the ϕ images for the T-cells show no distinguishable spatial pattern because the low A values result in ϕ values that are noise-dominated. Independent experiments with human platelets were repeated five times with

similar results. The T-cell images shown here were collected during a single experiment, but unpublished experiments with T-cells were previously repeated three times (using a previous iteration of MFM[1]) with similar results.

II. Supplementary Table 1 – Oligonucleotide sequences

Name	Sequence (5’-3’)
cRGDfK-presenting strand	5’-/5Hexynyl/-TTT GCT GGG CTA CGT GGC GCT CTT-/3AmMO/-3’
Anchor strand for glass	5’-/BHQ 1/-CGC ATC TGT GCG GTA TTT CAC TTT-/3Bio/-3’
Hairpin strand for glass	5’-GTG AAA TAC CGC ACA GAT GCG TTT GTA TAA ATG TTT TTT TCA TTT ATA C TTT AAG AGC GCC ACG TAG CCC AGC-3’
Opening strand	5’-GTA TAA ATG AAA AAA ACA TTT ATA C-3’
pMHC-presenting strand	5’-/5AmMC6/-CGC ATC TGT GCG GTA TTT CAC TTT-/3Bio/-3’
Anchor strand for AuNP	5’-/5ThiolMC6-D/-TTT GCT GGG CTA CGT GGC GCT CTT-/3BHQ_2/-3’
Hairpin for AuNP	5’-GTG AAA TAC CGC ACA GAT GCG TTT GTA TAA ATG TTT TTT TCA TTT ATA CTT TAA GAG CGC CAC GTA GCC CAG C-3’

The sequences are listed using Integrated DNA Technology (IDT)’s nomenclature. 3Bio = biotin ligated to the 3’ terminus. 5AmMC6 = 5’ amine ligated to the DNA via a 6-carbon linker. 3AmMO = amine linked to the 3’ DNA terminus. 5Hexynyl = 5’ terminal alkyne. 5ThiolMC6-D = protected thiol linked to the 5’ DNA terminus via a 6-carbon linker. BHQ 1 = Black Hole Quencher-1 attached to the 5’ DNA terminus. 3BHQ 2 = Black Hole Quencher 2 attached to the 3’ DNA terminus. Underlined letters denote self-complementary stem portions of hairpin strands.

III. Supplementary Table 2 – Comparison of high resolution TFM and MTFM techniques

	Study	Tension map spatial resolution*	Orientation resolvable?	Acquisition Time*	Required microscope	Note
**TFM techniques**	Sabass et al., 2008, Biophysical Journal[7]	1 µm	Yes	1 s	Confocal	Two color beads
	Colin-York et al., 2016, Nano Letters[8]	1-1.2 µm	Yes	20 s, 5 s	STED, Confocal	Modeling suggests 500 nm resolution is possible
	Colin-York et al., 2019, Nano Letters[9]	1 µm	Yes	10 s	SIM	Acquisition time increased by need to collect z-stack
	Stubb et al., 2020, Nano Letters[10]	1-2 µm	Yes	20 s	Spinning-disk confocal or widefield microscope	
**MTFM and MFM techniques**	Zhang et al., 2014 Nature Communications[6]	250 nm	No	0.1-1 s	Standard fluorescence microscope	Diffraction limited
	Brockman & Su et al., 2020 Nature Methods[1]	20 nm	No	1-10 min	Standard fluorescence microscope	DNA-PAINT based technique
	Brockman & Blanchard et al., Nature Methods[11]	200 nm	Yes**	3.6 s	Fluorescence polarization microscope	Original MFM paper
	This work, SIM-MFM	110 nm	Yes**	1 s	SIM	Orientation map has diffraction limited resolution

* Spatial resolution and acquisition time values shown represent our best estimates. Not all studies reported these values explicitly.

** In existing MFM techniques, orientation measurements have a two-fold degeneracy that prevents unique force vector mapping

IV. Supplementary Note 1 – Derivation of equations for analytical orientation measurement

To obtain a method for calculating force orientation analytically for each set of three intensity measurements (I_1, I_2, and I_3), we consider equations that can be used to represent fluorescence intensity:

$$I_1 = A\sin^2(\alpha_1 - \phi) + c \quad (1a)$$
$$I_2 = A\sin^2(\alpha_2 - \phi) + c \quad (1b)$$
$$I_3 = A\sin^2(\alpha_3 - \phi) + c \quad (1c)$$

where α is the polarization angle of the excitation beam, ϕ is the azimuthal angle of the force vector, and and A and c are scaling constants that are related to the background intensity, number of open tension probes, and the tilt angle of the force (θ). Because the three α angles are related to each other by rotations of $60°$, we can re-write these equations simply as:

$$I_1 = A\sin^2(\alpha_1 - \phi) + c \quad (2a)$$
$$I_2 = A\sin^2(\alpha_1 + 60° - \phi) + c \quad (2b)$$
$$I_3 = A\sin^2(\alpha_1 - 60° - \phi) + c \quad (2c)$$

Applying the half-angle formula yields:

$$I_1 = \frac{A}{2}(1 - \cos(2\alpha_1 - 2\phi)) + c \quad (3a)$$
$$I_2 = \frac{A}{2}(1 - \cos(2\alpha_1 + 120° - 2\phi)) + c \quad (3b)$$
$$I_3 = \frac{A}{2}(1 - \cos(2\alpha_1 - 120° - 2\phi)) + c \quad (3c),$$

and rearranging yields:

$$I_1 = \bar{I} - \frac{A}{2}\cos(2\alpha_1 - 2\phi) \quad (4a)$$
$$I_2 = \bar{I} - \frac{A}{2}\cos(2\alpha_1 + 120° - 2\phi) \quad (4b)$$
$$I_3 = \bar{I} - \frac{A}{2}\cos(2\alpha_1 - 120° - 2\phi) \quad (4c),$$

where

$$\bar{I} = \frac{A}{2} + c = \frac{I_1 + I_2 + I_3}{3} \quad (5)$$

Applying the identity $\cos(a + b) = \cos(a)\cos(b) + \sin(a)\sin(b)$ yields:

$$I_1 = \bar{I} - \frac{A}{2}(\cos(2\alpha_1)\cos(2\phi) + \sin(2\alpha_1)\sin(2\phi)) \quad (6a)$$
$$I_2 = \bar{I} - \frac{A}{2}(\cos(2\alpha_1 + 120°)\cos(2\phi) + \sin(2\alpha_1 + 120°)\sin(2\phi)) \quad (6b)$$
$$I_3 = \bar{I} - \frac{A}{2}(\cos(2\alpha_1 - 120°)\cos(2\phi) + \sin(2\alpha_1 - 120°)\sin(2\phi)) \quad (6c).$$

If we rotate our coordinate system such that $\alpha_1 = 0°$ and re-arrange, we obtain:

$$\bar{I} - I_1 = \frac{A}{2}\cos(2\phi) \qquad (7a)$$

$$\bar{I} - I_2 = \frac{A}{2}(\cos(120°)\cos(2\phi) + \sin(120°)\sin(2\phi)) \qquad (7b)$$

$$\bar{I} - I_3 = \frac{A}{2}(\cos(-120°)\cos(2\phi) + \sin(-120°)\sin(2\phi)) \qquad (7c).$$

Merging equations $(7a)$ and $(7b)$ yields:

$$\bar{I} - I_2 = (\bar{I} - I_1)\cos(120°) + \frac{A}{2}\sin(120°)\sin(2\phi) \qquad (8)$$

Re-arranging, we obtain

$$\frac{\bar{I} - I_2 - (\bar{I} - I_1)\cos(120°)}{\sin(120°)} = \frac{A}{2}\sin(2\phi) \qquad (9).$$

Dividing equation (9) by equation $(7a)$ rearranging yields

$$\tan(2\phi) = \frac{\bar{I} - I_2 - (\bar{I} - I_1)\cos(120°)}{(\bar{I} - I_1)\sin(120°)} \qquad (10)$$

and finally, rearranging the equation and adjusting the coordinate system to allow for arbitrary α_1 yields:

$$\phi = \frac{1}{2}\tan^{-1}\left(\frac{\bar{I} - I_2 - (\bar{I} - I_1)\cos(120°)}{(\bar{I} - I_1)\sin(120°)}\right) + \alpha_1 \qquad (11).$$

Now that ϕ is known, A can be solved for by re-arranging equation $(4a)$:

$$A = \frac{2(\bar{I} - I_1)}{\cos(2\alpha_1 - 2\phi)} \qquad (12).$$

Next, c can be obtained by re-arranging equation $(1a)$:

$$c = I_1 - A\sin^2(\alpha_1 - \phi) \qquad (13).$$

Finally, θ can be estimated using an equation that we previously derived:

$$\theta = \cos^{-1}\left(\sqrt{\frac{\frac{I_{max}}{c} - 0.069}{1 - 0.069}}\right) \qquad (14),$$

where

$$I_{max} = A + c \qquad (15).$$

V. Supplementary Note 2 – Examination of the effect of photobleaching on dynamic measurements of alignment

We tested whether photobleaching may contribute to the apparent increase in alignment. Specifically, photobleaching could cause certain regions of tension to drop out of the R calculation, resulting in an artificially-increased R after several consecutive SIM-MFM acquisitions. To evaluate this possibility, we simulated photobleaching by taking the timepoint of maximum area for each of the 22 increasing-alignment timelapses and re-measured R and T while gradually decreasing I_{avg} for the entire image (**Supplementary Fig 17a**). As expected, substantial levels of simulated photobleaching did artificially increase R, generally by ~25% (**Supplementary Fig 17b**). However, we found that this source of error should produce a significant negative correlation between R and T (**Supplementary Fig 17c** – Pearson's correlation coefficient=-0.38, p<0.001), and we observed no such correlation in the experimental results with the same set of platelets (**Supplementary Fig 17d**, p=0.07). Moreover, we conducted an analysis of photobleaching that suggests that 30 minutes of imaging (15 SIM-MFM acquisitions taken at 2-min intervals) should result in ~20-30% photobleaching and only cause ~15% of pixels to drop out of the automated analysis (**Supplementary Fig 18**). Based on our simulated photobleaching results, these small effects are not expected to cause substantial changes in the measured alignment parameter (**Supplementary Fig 17**). Taken together, these analyses suggest that, while photobleaching could partly contribute to the increase in R observed, the effect of photobleaching is modest at best and increasing alignment is driven predominantly by the platelets' phenotype as observed by integrin mechanics.

VI. Supplementary Note 3 – Description of analysis of T-cell receptor SIM-MFM data

When using SIM-MFM to image DNA hairpin tension probes opened by T-cell receptors (TCRs), we found that T-cells did not exhibit any notable polarization-dependent response. To quantify this finding, we measured the ratio $\langle A\rangle/(\langle c\rangle + 1)$ under cells and in the background. As a control, we also quantified $\langle A\rangle/(\langle c\rangle + 1)$ for platelets. As stated in the main text, A and c are measures of the fit sinusoid for a given pixel such that A is a measure of the polarization-dependent fluorescence intensity signal and c is a measure of the polarization-independent fluorescence intensity signal – see **Supplementary Fig 19**. The angle brackets $\langle\ \rangle$ denote ensemble averaging, meaning we averaged all recorded A and c values measured under the cell and in the local background near the cell. Because the intensity values are background-subtracted we added 1 to $\langle c\rangle$ to prevent instabilities in the estimate of the polarization ratio. Higher $\langle A\rangle/(\langle c\rangle + 1)$ means that the polarization-dependent component of fluorescence intensity is strong, indicating the presence of temporally stable forces with a substantial in-plane component ($\theta \gg 0°$). Conversely, low $\langle A\rangle/(\langle c\rangle + 1)$ means that the polarization dependent component of fluorescence intensity is weak, which can have multiple interpretations discussed below.

While platelets exhibited large $\langle A\rangle/(\langle c\rangle + 1)$ values (median of ~0.24), T-cells exhibited a low $\langle A\rangle/(\langle c\rangle + 1)$ (median of ~0.1) – see **Fig. 6c**. Measurement of $\langle A\rangle/(\langle c\rangle + 1)$ values in the cell-free background for both cell types exhibited values similar to that measured under T-cells (median ~0.1). Inspection of maps of A and c show that virtually all T-cell signal is polarization-independent (**Figs. 6**, S17). Together, these results suggest that tension is not oriented in a manner that results in the polarization response needed for MFM to map orientation.

VII. REFERENCES

1 Brockman, J. M., Blanchard, A. T., *et al.* Mapping the 3D orientation of piconewton integrin traction forces. *Nat. Methods* **15**, 115, doi:10.1038/nmeth.4536 (2017).

2 Descloux, A., Grußmayer, K. S. & Radenovic, A. Parameter-free image resolution estimation based on decorrelation analysis. *Nat. Methods* **16**, 918-924, doi:10.1038/s41592-019-0515-7 (2019).

3 Ball, G. *et al.* SIMcheck: a Toolbox for Successful Super-resolution Structured Illumination Microscopy. *Sci. Rep.* **5**, 15915, doi:10.1038/srep15915 (2015).

4 Mardia, K. V., Jupp, P. E., *Directional Statistics* 13-24 (Wiley, 1999).

5 Zhu, C. & Chen, W. in *Single-molecule Studies of Proteins* 77-96 (Springer New York, 2013).

6 Zhang, Y., Ge, C., Zhu, C. & Salaita, K. DNA-based digital tension probes reveal integrin forces during early cell adhesion. *Nat. Commun* **5**, 5167, doi:10.1038/ncomms6167 (2014).

7 Sabass, B., Gardel, M. L., Waterman, C. M. & Schwarz, U. S. High resolution traction force microscopy based on experimental and computational advances. *Biophys. J.* **94**, 207-220, doi:10.1529/biophysj.107.113670 (2008).

8 Colin-York, H. *et al.* Super-Resolved Traction Force Microscopy (STFM). *Nano Lett.* **16**, 2633-2638, doi:10.1021/acs.nanolett.6b00273 (2016).

9 Colin-York, H. *et al.* Spatiotemporally Super-Resolved Volumetric Traction Force Microscopy. *Nano Lett.* **19**, 4427-4434, doi:10.1021/acs.nanolett.9b01196 (2019).

10 Stubb, A. *et al.* Fluctuation-Based Super-Resolution Traction Force Microscopy. *Nano Lett.* **20**, 2230-2245, doi:10.1021/acs.nanolett.9b04083 (2020).

11 Brockman, J. M. *et al.* Live-cell super-resolved PAINT imaging of piconewton cellular traction forces. *Nat. Methods*, doi:10.1038/s41592-020-0929-2 (2020).
