# Supplementary material for: Turn-key mapping of cell receptor force orientation and magnitude using a commercial structured illumination microscope
Source: Nat Commun. 2021 Aug 3;12:4693. doi: 10.1038/s41467-021-24602-x (PMC8333341; doi:10.1038/s41467-021-24602-x)
Supplement: Supplementary file 2 — Reporting Summary [file 41467_2021_24602_MOESM2_ESM.pdf]

nature research

Corresponding author(s): Khalid Salaita

Last updated by author(s): 06/10/2021

Reporting Summary

Nature Research wishes to improve the reproducibility of the work that we publish. This form provides structure for consistency and transparency in reporting. For further information on Nature Research policies, see our Editorial Policies and the Editorial Policy Checklist.

Statistics

For all statistical analyses, confirm that the following items are present in the figure legend, table legend, main text, or Methods section.

n/a	Confirmed	
☐	☒	The exact sample size (n) for each experimental group/condition, given as a discrete number and unit of measurement
☐	☒	A statement on whether measurements were taken from distinct samples or whether the same sample was measured repeatedly
☐	☒	The statistical test(s) used AND whether they are one- or two-sided<br>*Only common tests should be described solely by name; describe more complex techniques in the Methods section.*
☒	☐	A description of all covariates tested
☐	☒	A description of any assumptions or corrections, such as tests of normality and adjustment for multiple comparisons
☐	☒	A full description of the statistical parameters including central tendency (e.g. means) or other basic estimates (e.g. regression coefficient) AND variation (e.g. standard deviation) or associated estimates of uncertainty (e.g. confidence intervals)
☐	☒	For null hypothesis testing, the test statistic (e.g. F, t, r) with confidence intervals, effect sizes, degrees of freedom and P value noted<br>*Give P values as exact values whenever suitable.*
☒	☐	For Bayesian analysis, information on the choice of priors and Markov chain Monte Carlo settings
☒	☐	For hierarchical and complex designs, identification of the appropriate level for tests and full reporting of outcomes
☐	☒	Estimates of effect sizes (e.g. Cohen's d, Pearson's r), indicating how they were calculated

Our web collection on statistics for biologists contains articles on many of the points above.

Software and code

Policy information about availability of computer code

Data collection	Images were collected using Nikon Elements v5.0.2, which has modules that can be used for automated 3D-SIM and 2D-SIM acquisitions.
Data analysis	All simulations and most computational analyses were performed in MATLAB 2018b or later. See supplemental software for representative analysis code. Super-resolution reconstruction was performed with Nikon Elements software (v5.2.1), and evaluated in Fiji v1.53c (https://imagej.net/Fiji) with the SIMcheck plugin v1.3 (https://imagej.net/SIMcheck). Resolution enhancement was performed with the MATLAB version of the ImDecorr ImageJ plugin v.1.1.8. (https://github.com/Ades91/ImDecorr/tree/master/ijplugin).

For manuscripts utilizing custom algorithms or software that are central to the research but not yet described in published literature, software must be made available to editors and reviewers. We strongly encourage code deposition in a community repository (e.g. GitHub). See the Nature Research guidelines for submitting code & software for further information.

Data

Policy information about availability of data

All manuscripts must include a data availability statement. This statement should provide the following information, where applicable:

- Accession codes, unique identifiers, or web links for publicly available datasets
- A list of figures that have associated raw data
- A description of any restrictions on data availability

Source data are provided with this paper. Raw SIM acquisitions, with corresponding background illumination correction images, of platelets (Figs. 1-2, 6, S3-7, S9, S12-13, S19), 3T3 Fibroblasts (Figs. 3, S8-9) T-cells (Figs. 6, S19), as well as platelet timelapses (Figs. 5, S14, S16-18), are freely available at https://doi.org/10.15139/S3/FXOHWV. Additional datasets generated during the current study (i.e. processed data files generated by MATLAB code) are available from the corresponding author on reasonable request. Representative raw data from a SIM-MFM acquisition, as well as raw data used for illumination profile correction, are included with the supplemental software attached to this manuscript.

Field-specific reporting

Please select the one below that is the best fit for your research. If you are not sure, read the appropriate sections before making your selection.

- [x] Life sciences
- [ ] Behavioural & social sciences
- [ ] Ecological, evolutionary & environmental sciences

For a reference copy of the document with all sections, see nature.com/documents/nr-reporting-summary-flat.pdf

Life sciences study design

All studies must disclose on these points even when the disclosure is negative.

Sample size	Sample size was determined strictly by availability; we generally included all high signal-to-noise images and time-lapses collected across multiple experiments in our analyses.
Data exclusions	Images were only collected of cells that exhibited high signal-to-noise tension fluorescence signal. Low-signal cells were excluded because 1) they often could not be seen when scanning the surface, 2) they were generally interpreted as exhibiting anomalous spreading or potentially having completed the process of spreading and exhaustion, and 3) they would not have been interpretable using many of the analyses presented in this paper.
Replication	Experiments with platelets and fibroblasts were repeated at least 3 independent times with independent batches of cells (from different donors, in the case of platelets) and independent surface preparations. We found that cells spreading on high-quality surfaces and imaged using a properly aligned microscope exhibited consistent behaviors across experimental replicates. Experiments with T-cells presented in this work were performed using T-cells harvested from a single mouse on five separate surfaces. Because these (largely inconclusive) findings agreed well with unpublished results from ~3 years ago, we chose not to repeat T-cell experiments to avoid unnecessarily sacrificing more mice. To our recollection, all experimental replicates were successful. However, in MTFM and MFM studies, failed experiments are generally interpreted as resulting from 1) failed surface preparation, resulting in low or heterogeneous tension probe surface density, 2) the use of old mice for T-cell harvesting or improper handeling of T-cells, 3) seeding of cells at surface densities that are too high (this is most commonly an issue with platelets) or 4) improper timing during experimentation (3T3 cells and platelets generally need ~10 minutes to contact the surface and spread, and exhaust themselves and degrade the surface within tens of minutes).
Randomization	Samples were generally not separated into groups. The one exception is that the temporal analysis of platelet dynamics involved post-hoc separation of platelets into groups of increasing-alignment and non-increasing-alignment using objective methods clearly outlined in the text.
Blinding	Blinding was not relevant to the experimental design because comparisons were not made between groups. Experiments with human platelets experiments were also not blinded because platelet donation was coordinated by the investigators conducting the experiments.

Reporting for specific materials, systems and methods

We require information from authors about some types of materials, experimental systems and methods used in many studies. Here, indicate whether each material, system or method listed is relevant to your study. If you are not sure if a list item applies to your research, read the appropriate section before selecting a response.

Materials & experimental systems

n/a	Involved in the study	
x		Antibodies
	x	Eukaryotic cell lines
x		Palaeontology and archaeology
	x	Animals and other organisms
	x	Human research participants
x		Clinical data
x		Dual use research of concern

Methods

n/a	Involved in the study	
x		ChIP-seq
x		Flow cytometry
x		MRI-based neuroimaging

Eukaryotic cell lines

Policy information about cell lines

Cell line source(s)	Mouse embryonic fibroblast cells (NIH 3T3s) stably expressing GFP-vinculin were a gift from the lab of Prof. Andres Garcia (originally generated in the lab of Andres Garcia).
Authentication	The cell lines have not been authenticated.
Mycoplasma contamination	The cell lines were not tested for mycoplasma contamination.
Commonly misidentified lines (See ICLAC register)	No commonly misidentified cell lines were used.

Animals and other organisms

Policy information about studies involving animals; ARRIVE guidelines recommended for reporting animal research

Laboratory animals	In our studies, male and female OT1 transgenic mice are used equally between the ages of 6-8 weeks.
Wild animals	This study did not involve the use of wild animals
Field-collected samples	This study did not involve any field-collected samples
Ethics oversight	All animals were housed and bred at the Division of Animal Resources at Emory University, in accordance with the Institutional Animal Care and Use Committee (IACUC) protocol (PROTO201800239) of the IACUC of Emory University.

Note that full information on the approval of the study protocol must also be provided in the manuscript.

Human research participants

Policy information about studies involving human research participants

Population characteristics	For platelet studies, four males (age 25-40) and one female (age 25-40) were used in this study.
Recruitment	Participants were selected as willing volunteers according primarily to convenience. Participants were acquaintances of the investigators who were contacted and asked to donate platelets on the day of or prior to experimentation. Because no pathologies were investigated, the participants, who self-identified as not having any platelet-related illnesses, were assumed to represent a standard population. However, the small sample size and
Ethics oversight	All procedures using donor-derived human platelets were approved by the Institutional Review Board of Children's Healthcare of Atlanta/Emory University (IRB # IRB00006228). Written, informed consent was received from participants prior to their inclusion in studies.

Note that full information on the approval of the study protocol must also be provided in the manuscript.
